# Post-transcriptional regulation in cranial neural crest cells expands developmental potential

**DOI:** 10.1073/pnas.2212578120

**Published:** 2023-02-01

**Authors:** Rachel A. Keuls, Young Sun Oh, Ivanshi Patel, Ronald J. Parchem

**Affiliations:** ^a^Development, Disease Models & Therapeutics Graduate Program, Baylor College of Medicine, Houston, TX 77030; ^b^Stem Cells and Regenerative Medicine Center, Baylor College of Medicine, Houston, TX 77030; ^c^Center for Cell and Gene Therapy, Baylor College of Medicine, Houston, TX 77030; ^d^Department of Neuroscience, Baylor College of Medicine, Houston, TX 77030; ^e^Department of Molecular and Cellular Biology, Baylor College of Medicine, Houston, TX 77030; ^f^Program in Developmental Biology, Baylor College of Medicine, Houston, TX 77030

**Keywords:** multipotency, pluripotency, single-cell, microRNAs, chromatin accessibility

## Abstract

Developmental potential is progressively restricted after germ layer specification during gastrulation. However, cranial neural crest cells challenge this paradigm, as they develop from anterior ectoderm, yet give rise to both ectodermal derivatives of the peripheral nervous system and ectomesenchymal bone and cartilage. How cranial neural crest cells differentiate into multiple lineages is poorly understood. Here, we demonstrate that cranial neural crest cells possess a transient state of increased chromatin accessibility. We profile the spatiotemporal emergence of premigratory neural crest and find evidence of lineage bias toward either a neuronal or ectomesenchymal fate, with each expressing distinct factors from earlier stages of development. We identify the miR-302 miRNA family to be highly expressed in cranial neural crest cells and genetic deletion leads to precocious specification of the ectomesenchymal lineage. Loss of *mir-302* results in reduced chromatin accessibility in the neuronal progenitor lineage of neural crest and a reduction in peripheral neuron differentiation. Mechanistically, we find that *mir-302* directly targets *Sox9* to slow the timing of ectomesenchymal neural crest specification and represses multiple genes involved in chromatin condensation to promote accessibility required for neuronal differentiation. Our findings reveal a posttranscriptional mechanism governed by miRNAs to expand developmental potential of cranial neural crest.

## Significance

Craniofacial birth defects are one of the most common congenital malformations in humans and are caused by defects in neural crest cells. In early vertebrate development, cranial neural crest cells acquire the ability to differentiate into many different cell types to generate the craniofacial structure and peripheral nervous system. To better understand how neural crest are endowed with expanded capacity to generate cells of multiple lineages, we use single-cell multiomic approaches to uncover a role for posttranscriptional regulation to expand chromatin accessibility during the earliest stages of neural crest specification. Our findings provide insight into ways that stem cells can increase their ability to differentiate into multiple cell types, which may improve our ability to generate tissues for regenerative medicine.

Repairing tissues and organs to restore lost function due to aging, disease, and injury is a major goal for regenerative medicine, one that relies on the ability to differentiate patient cells across lineages. To successfully generate cells and tissues for human patients, much research has focused on reprogramming cells to a pluripotent state before directing their differentiation toward the desired cell type. As cells transition out of pluripotency during gastrulation, they are specified to one of three primary germ layers: endoderm, mesoderm, or ectoderm. Once germ layer specification has occurred, cells can typically only differentiate into derivates of their germ layer of origin. Cranial neural crest cells, however, are a transient population of multipotent precursors that are specified from anterior ectoderm yet expand their potential to generate both mesodermal and ectodermal derivatives. Premigratory neural crest cells will undergo epithelial-to-mesenchymal transition (EMT) and migrate to distant sites within the embryo, where they terminally differentiate and give rise to ectomesenchymal derivatives contributing to the craniofacial skeleton and ectodermal lineages that contribute to the peripheral nervous system (1–5). Understanding how cranial neural crest differentiate across germ layers will advance the field of regenerative medicine.

Single-cell analysis of embryonic day (E) 8.5 and E9.5 mouse embryos suggested that cranial neural crest cells have high transcriptional similarity during delamination and that upregulation of gene expression associated with different fate choices occurs during migration (3). However, single-cell analysis of Wnt1-Cre+ neural crest prior to E8.5 in mouse revealed that Oct4 was reactivated in a subset of premigratory neural crest progenitors and was required for differentiation of the ectomesenchymal lineage of derivatives but not neuronal derivatives (6). Indeed, early studies using histological approaches suggested distinct populations of cranial neural crest at the end of gastrulation (7). For example, analysis of four somite stage mouse embryos revealed a majority of ectomesenchymal neural crest are specified at the lateral nonneural domain whereas neural crest cells of eight somite stage embryos were specified in a more medial neural domain (7). How these distinct premigratory neural crest are specified and whether they share similar mechanisms of fate specification remain to be determined.

Other work has focused on the maintenance or reactivation of transcripts associated with pluripotency from earlier stages of development. Studies in *Xenopus* suggested that cranial neural crest cells retain expression of genes from earlier stages of development to expand their potential (8). This work was supported by evidence of an avian neural crest stem cell niche that expressed both pluripotency and canonical neural crest transcription factors (9). In response to folic acid, folate receptor alpha translocates into the nucleus and activates transcription of *Oct4*, *Sox2* and *Klf4* in a mouse cranial neural crest cell line (10). Similarly, reactivation of Oct4 and Nanog in neural crest cells have been proposed in an alternate model of reprogramming, which is important for differentiation of the ectomesenchymal, but not neuronal lineage of neural crest (6, 11). However, studies of early neural crest development have identified specified neural crest in gastrula stage chick (12) and rabbit embryos (13). Recent studies using explants from blastula stage chick embryos have shown specification of neural crest prior to segregation of neuroectodermal and mesodermal lineages (14). Similarly, studies of human embryonic stem cell differentiation into neural crest suggest specification from precursors independent of ectoderm or mesoderm derivation (15, 16). Consistent with progressive differentiation, human neural crest cell differentiation and single-cell transcriptomic studies in *Xenopus* suggested restricted fate of neural crest cells and little retention of gene expression, as pluripotent blastula cells differentiate into cranial neural crest (16, 17). Thus, it remains unclear how premigratory cranial neural crest populations use factors expressed in earlier stages of development to promote differentiation potential.

Developmental potential is defined by specific gene regulatory networks, which incorporate chromatin structure, transcription factors, regulatory elements, and posttranscriptional regulators. Analysis of intermediate phases during somatic cell reprogramming has demonstrated that a transient state of chromatin hyperaccessibility poises pluripotency loci for subsequent activation (18–20). Previously, a class of small RNAs known as microRNAs (miRNAs) were demonstrated to induce a permissive chromatin structure allowing for the reprogramming of fibroblasts to a neuronal identity (21). Similarly, ectopic introduction of miRNAs enriched in mouse and human embryonic stem cells is sufficient to reprogram somatic cells to an induced pluripotent state (22–24). How chromatin structure is regulated within cranial neural crest as they acquire the ability to differentiate into multiple lineages remains to be explored. Furthermore, whether miRNAs regulate the acquisition of developmental potential of cranial neural crest has not been studied.

We hypothesized that changes in chromatin accessibility are regulated by miRNAs to promote the expansion of developmental potential in cranial neural crest. Our results demonstrate a transient increase in chromatin accessibility concomitant with gene expression bias toward a neuronal or ectomesenchymal fate at the neural plate border in mice. Our results suggest that transcription factors associated with early fate specification within the epiblast regulate the formation of premigratory neural crest cells. Functionally, the miR-302 family targets *Sox9* to prevent precocious formation of ectomesenchymal derivatives and chromatin remodelers to promote chromatin accessibility required for differentiation of peripheral neurons. Our findings reveal distinct post-transcriptional mechanisms that expand developmental potential of murine cranial neural crest cells.

## Results

### Chromatin Accessibility Increases during Cranial Neural Crest Specification.

**Fig. 1.**
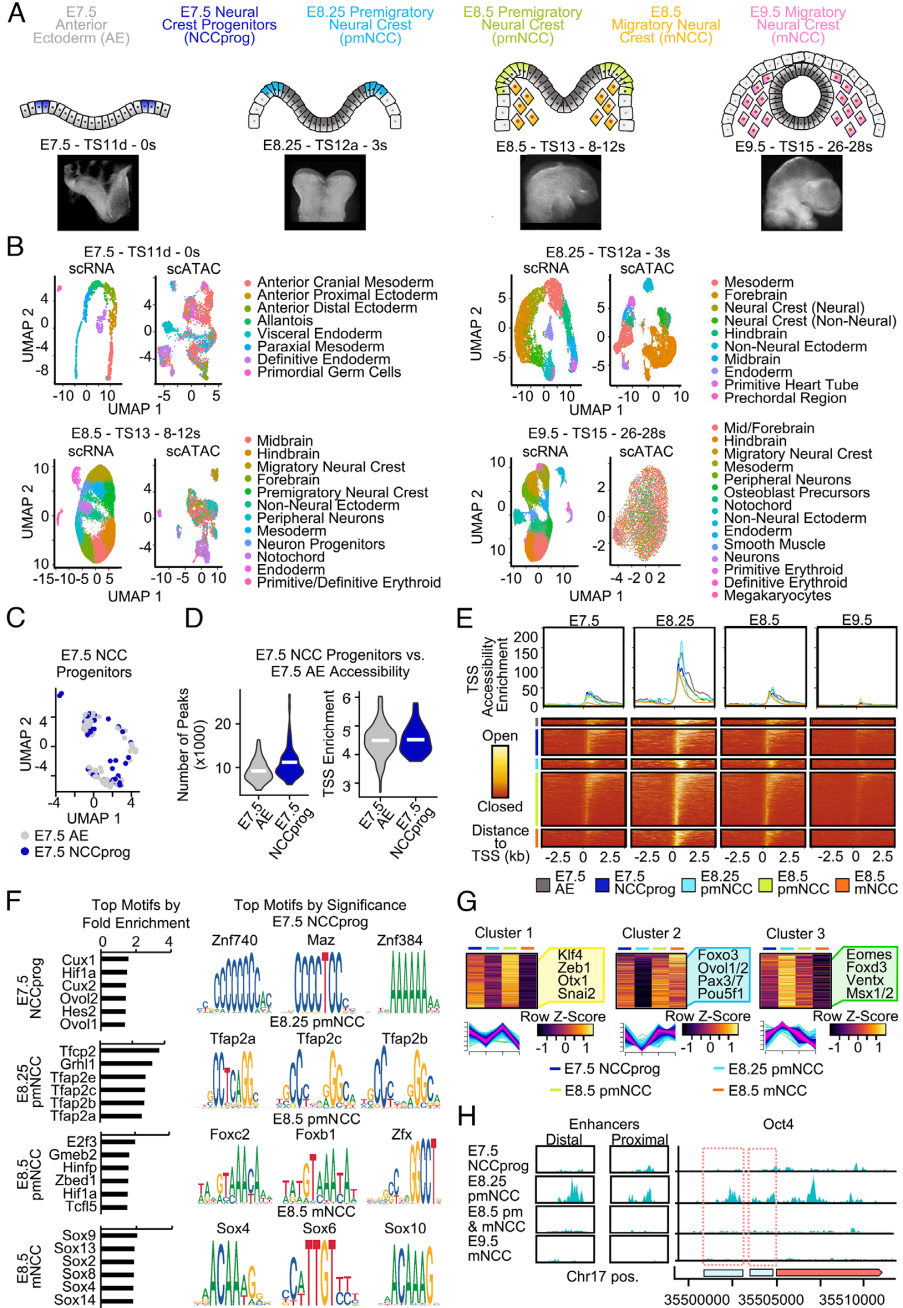
Chromatin accessibility increases during cranial neural crest specification. (*A*) Embryos were dissected at E7.5 (TS11d, 0 somites), E8.25 (TS12a, 3 somites), E8.5 (TS13, 8 to 12 somites), and E9.5 (TS15, 26 to 28 somites), and both single-cell ATAC and scRNA-seq were performed on the epiblast and mouse cranial region using 10X Genomics to assess chromatin structure and gene expression changes during cranial neural crest formation. (*B*) Uniform Manifold Approximation and Projection for Dimension Reduction (UMAP) plots showing cell populations obtained from single-cell ATAC and scRNA-seq at each stage. Population-specific genes included within Dataset S1. (*C*) E7.5 Neural crest progenitors (NCCprog, 50 cells) expressing Sox9, Pax7, Pax3, or Msx1 were identified from cells of the E7.5 anterior ectoderm (AE, 163 cells). (*D*) Number of peaks and TSS enrichment in cells of the E7.5 anterior ectoderm compared with the neural crest progenitors. (*E*) Peak plot and heat map showing accessibility over time of differentially accessible peaks in comparing neural crest-associated populations at E7.5, E8.25, and E8.5 to nonneural crest at respective stages. All significantly differentially accessible peaks are included in Dataset S2. (*F*) Top enriched motifs in differentially accessible peaks of each neural crest population. Bar plots contain the top five motifs by fold change, while the motif sequence logo is shown for the top three most significant motifs. All significantly enriched peaks in the differentially accessible peaks are included in Dataset S2. The presence of motifs for top factors was analyzed in all peaks and is included in Dataset S3 (0 mismatches allowed) and in Dataset S4 (one mismatch allowed). (*G*) Clustering analysis was used to group motifs based on their accessibility in E7.5 neural crest progenitors, E8.25 premigratory neural crest, E8.5 premigratory neural crest, and E8.5 migratory neural crest. All clustering data included within Dataset S9. (*H*) Coverage plot revealing increased accessibility of Oct4 locus and regulatory elements in E8.5 neural crest cells.

To characterize changes in chromatin accessibility and transcriptomics during the specification of cranial neural crest, we performed single-cell assay for transposase-accessible chromatin sequencing (scATAC-seq) and single-cell mRNA sequencing (scRNA-seq) of the mouse epiblast at E7.5, and the cranial region at E8.25, E8.5, and E9.5 (Fig. 1 *A* and *B*). Latent semantic indexing analysis was performed on the count matrix normalized by term frequency inverse document frequency (TF-IDF) followed by singular value decomposition (SVD) to correct for differences in cellular sequencing depth, across peaks and to integrate datasets. Cell populations were defined by gene expression using the markers in *SI Appendix*, Table S1, and chromatin data were overlaid as previously described (25). Population-specific genes for all cell populations at each age are included within Dataset S1.

Previous studies have shown early expression of neural crest genes in the neural folds of presomitic embryos (26–28) and SOX9-positive cells at the neural plate border of two somite embryos (7). Therefore, we identified neural crest progenitors that express at least one neural crest specification factor (*Sox9*, *Pax3*, *Pax7*, or *Msx1*) from anterior ectoderm (expressing *Sox2*, *Otx2*, *Gas1*, *Bcat1, Lefty2, Fgf5*) of a presomitic embryo (TS11d) at E7.5. To determine how chromatin accessibility changes during specification of the earliest neural crest progenitors, we compared accessibility between the E7.5 anterior ectoderm and E7.5 neural crest progenitors (Fig. 1*C*). We found that the number of peaks increased slightly in E7.5 neural crest progenitors as compared with the E7.5 anterior ectoderm, whereas transcriptional start site (TSS) accessibility was similar (Fig. 1*D*). At later stages, neural crest were identified using canonical markers such as *Sox9*, *Pax3*, *Pax7*, and *Tfap2a*. Migratory neural crest were distinguished from premigratory neural crest using *Sox10* as a marker specific for migratory cells (29, 30). Other studies have noted expression of Sox10 in neural crest cells at the neural plate border just prior to the onset of delamination (13, 31), therefore our migratory neural crest populations may be inclusive of some premigratory cells. To characterize changes in chromatin accessibility during neural crest development, we first analyzed global chromatin accessibility across all peaks and found that chromatin accessibility increased from E7.5 to E8.25 (*SI Appendix*, Fig. S1 *A* and *B*). At E8.5, global chromatin accessibility was similar to E7.5 and then subsequently declined at E9.5 (*SI Appendix*, Fig. S1 *A* and *B*). Next, we identified differentially accessible peaks and enriched motifs in neural crest cells from E7.5 to E9.5 and found greater levels of open chromatin from E7.5 to E8.25, which was lost by E9.5, supporting a unique mechanism of increased accessibility associated with neural crest specification (Fig. 1*E*; all differentially accessible peaks available in Dataset S2). Nucleosome signal, number of peaks, TSS enrichment, and the number of transcripts in E8.25 and E8.5 neural crest populations were similar to E7.5 neural crest progenitors (*SI Appendix*, Fig. S1*C*). Enriched motifs in the E7.5 neural crest precursors included genes associated with gastrulation such as Ovol1/2, Maz and zinc finger transcription factors such as Znf740 and Znf384 (Fig. 1*F*; all significantly enriched motifs included for each neural crest-associated population in Dataset S2). Top enriched motifs in E8.25 premigratory neural crest included canonical neural crest specification factors including Tfap2a/b/c, whereas top enriched motifs in E8.5 premigratory neural crest included Foxb/c. In E8.5 migratory neural crest, enrichment included multiple groups of Sox transcription factors such as Sox2, Sox8, Sox9, Sox10, and others (Fig. 1*F*). The presence of motifs of a top factor at each age was analyzed in all peaks and is included in Dataset S3 (0 motif mismatches) and Dataset S4 (one motif mismatch allowed). Motifs for canonical neural crest specification factors such as Pax7 are enriched in differentially accessible peaks at each stage of neural crest development (Dataset S2). These findings are in line with previous results (32–35) and highlight changes in chromatin landscape and identify regulatory elements enriched during mouse neural crest development.

Previous studies found chromatin hyperaccessibility during somatic cell reprogramming, which poised embryonic stem cell loci for subsequent expression (21). Thus, we hypothesized that increased chromatin accessibility would facilitate the use of multiple gene regulatory networks in cranial neural crest cells. Therefore, we analyzed how peaks and motifs are shared between neural crest cells across developmental time. Interestingly, when we compared motifs enriched in bona fide E8.25 and E8.5 neural crest cells with the E7.5 neural crest progenitors, we found that the majority of motifs were shared (*SI Appendix*, Fig. S1*D*; quantification for each comparison available in Dataset S5; unique and shared peaks and motifs from comparisons between each E8.25 to E8.5 neural crest population and the E7.5 neural crest progenitors available in Dataset S6; unique and shared peaks and motifs from sequential comparisons between neural crest-associated populations across developmental time available in Dataset S7). We found that peaks and shared motifs between the E7.5 neural crest progenitors and the E8.25 premigratory neural crest were predicted to be involved in post transcriptional regulation by miRNAs, suggesting an important role for posttranscriptional regulation as cranial neural crest are specified (*SI Appendix*, Fig. S1*E*). Peaks shared included genes such as Chd7, Zfp652, and Lmntd1, while shared motifs included Klf1/2/4, Zic3, Prrx2, Tfap2a, and Snai1 (Fig. 1*E*). We next determined shared peaks and motifs between the E7.5 neural crest progenitors and E8.5 premigratory neural crest and found that shared peaks were predicted to be involved in the negative regulation of stem cell differentiation, cell proliferation, and migration and included early neural crest specification and neural plate border genes such as Pax7 and Msx1 (*SI Appendix*, Fig. S1*E*). Motifs shared between the E7.5 neural crest progenitors and E8.5 premigratory neural crest cells were predicted to be involved in the formation of neural crest derivatives and miRNA function and included genes such as Klf4, Ventx, Pax3, Foxd3, and Pou3f1/3. Next, we identified motifs enriched in regions of chromatin accessibility shared between the E7.5 neural crest progenitors and populations of neural crest at E8.25 and E8.5 (*SI Appendix*, Fig. S1*F* and Dataset S8). Motifs for factors such as Sox9, Sox2, and Tfap2a were enriched within peaks shared between the E7.5 neural crest progenitors and the E8.25 premigratory neural crest. Within peaks shared between the E7.5 neural crest progenitors and the E8.5 migratory neural crest included factors such as Gata3, Ets, and Ronin (*SI Appendix*, Fig. S1*F* and Dataset S8). These results suggest that early presomitic neural crest populations have shared epigenetic landscape with the anterior ectoderm and that accessibility of regulatory elements is similar between E8.25 and E8.5 neural crest cells and presomitic neural crest progenitors at E7.5.

To analyze how motif accessibility changed during neural crest specification, we performed unsupervised clustering of motifs that were significantly enriched in differentially accessible peaks of the E7.5 neural crest progenitors, E8.25 premigratory, E8.5 premigratory, and E8.5 migratory neural crest cells (Fig. 1*G* and Dataset S9). Cluster 1 represented motifs that were accessible in the E7.5 neural crest progenitors, E8.5 premigratory neural crest, and more modestly accessible in the E8.25 premigratory neural crest. Cluster 1 motifs included factors such as Klf4, Zeb1, Otx1, and Snai2 (Fig. 1*G*). Cluster two motifs were accessible in each neural crest population except the E8.25 premigratory neural crest and contained factors such as Oct4, Foxo3, Ovol1/2, and Pax3/7. Cluster 3 motifs were most accessible in the E8.25 premigratory neural crest and contained motifs for factors such as Eomes, Ventx, Foxd3, and Msx1/2 (Fig. 1*G*). We next analyzed how accessibility around pluripotency loci changes during neural crest specification. Analysis of the Oct4 locus revealed accessibility from E7.5 through E8.5, yet accessibility was increased in E8.25 premigratory neural crest around enhancers used in both naïve and primed pluripotency (Fig. 1*H*). Together these data suggest that a transient increase in chromatin accessibility is present during specification of cranial neural crest cells.

### Emergence of Ectomesenchymal and Neuronal-Biased Neural Crest.

**Fig. 2.**
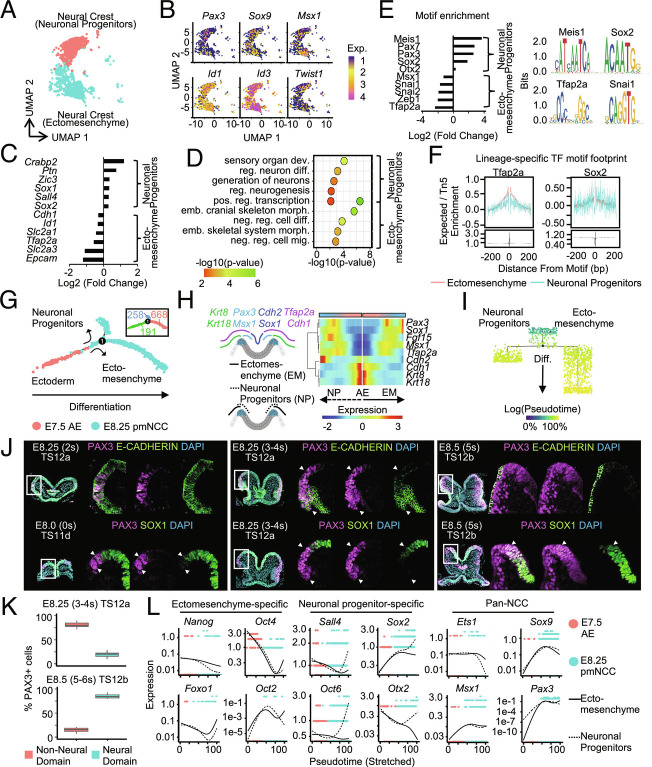
Emergence of ectomesenchymal and neuronal-biased neural crest. (*A*) UMAP plot showing separation between the two distinct populations of premigratory neural crest at E8.25. (*B*) UMAP plots of early neural crest specification factors showing expression in both populations. (*C*) Differentially expressed genes in each population of premigratory neural crest. All population-specific genes included in Dataset S10. (*D*) Gene ontology analysis of differentially expressed genes reveals a fate bias of the earliest premigratory neural crest. All gene ontology terms included for each population in Dataset S11. (*E*) Significantly enriched motifs were identified from differentially accessible peaks of each E8.25 premigratory neural crest population. Representative motifs are shown for each lineage and all differentially accessible peaks and significantly enriched motifs included in Dataset S12. (*F*) Transcription factor motif footprint for Tfap2a and Sox2 showing enrichment in the ectomesenchymal and neuronal lineages, respectively. (*G*) Pseudotime trajectory of the E7.5 anterior ectoderm and the E8.25 premigratory cranial neural crest reveals two distinct populations. Inset shows number of cells in each cell state. Branched expression analysis modeling included in Dataset S13. Genes enriched in E8.25 premigratory neural crest that group with cells of the E7.5 anterior ectoderm to the left of the node are included in Dataset S14. (*H*) Branched pseudotime heatmap showing expression of medial/lateral genes. (*I*) Differentiation progression of E7.5 anterior ectoderm and E8.25 cranial neural crest populations. Cells are colored according to their progression in pseudotime. Cells of the ectomesenchyme were further along the differentiation trajectory compared with neuronal progenitors. (*J*) Zoomed-in view of the neural plate border showing immunofluorescence of PAX3-positive cells forming within the E-CADHERIN- and SOX1-positive domains at E8.0, E8.25, and E8.5. (Wide field view of cross-sections with both individual and merged channels, and full time course available in *SI Appendix*, Fig. S2*D*). (*K*) Quantification of the number of neural crest that form as part of the nonneural domain and the neural domain at both E8.25 and E8.5. Significantly more neural crest form as a part of the lateral ectomesenchymal E-CADHERIN-positive domain compared with the medial E-CADHERIN-negative neural region at E8.25 (ANOVA *P* = 2.09e-08). At E8.5 significantly more neural crest form as a part of the medial SOX1-positive neural domain compared with the lateral nonneural Sox1-negative domain (ANOVA *P* = 3.57e-06). Quantifications included in Dataset S15. (*L*) Branched pseudotime plot showing lineage-specific expression of factors expressed in early development. Cells are plotted based on their expression of each marker, ordered in pseudotime, and colored by population.

To interrogate changes in gene expression and chromatin accessibility during specification of premigratory neural crest, we further analyzed scRNA-seq and scATAC-seq of the E8.25 cranial region. Cell clustering revealed two distinct populations of premigratory cranial neural crest cells that robustly express canonical neural crest transcription factors (Fig. 2 *A* and *B*). Since previous studies suggested that specification of cranial neural crest within different domains of the ectoderm may have differential capacity to give rise to derivatives (7), we identified differentially expressed genes and used biological gene ontology analysis to identify differences between the two populations. Within one population of premigratory neural crest, we identified enriched genes such as *Tfap2a, E-cadherin*, and *Epcam*, that were predicted to be involved in cranial skeleton morphogenesis, and we termed this population ectomesenchyme (Fig. 2 *C* and *D*; full differential expression analysis provided in Dataset S10 and full gene ontology analysis provided in Dataset S11). Within the other population of premigratory neural crest, we identified differentially expressed genes, such as *Sox1*, *Zic2*, and *Sall4,* that were predicted to be involved in neurogenesis, and we designated this population as neuronal progenitors (Fig. 2 *C* and *D*). Next, we identified differentially accessible peaks and significantly enriched motifs, and in agreement with a lineage specific function, we identified motifs for Tfap2a, and Snai2 enriched within ectomesenchyme, whereas the neuronal lineage was enriched for motifs of Pax3/7, Sox2, and Otx2 (Fig. 2*E*; differential peaks and enriched motif analysis provided in Dataset S12). Similarly, analysis of motif footprints for these lineage-specific transcription factors identified an enrichment within their respective lineages. For example, Tfap2a was enriched within the ectomesenchymal lineage, and Sox2 was enriched within the neuronal lineage (Fig. 2*F*).

To characterize the emergence of each premigratory neural crest lineage, we aligned the cells of the E7.5 anterior epiblast and E8.25 premigratory cranial neural crest along a pseudotime trajectory. Premigratory neural crest reach a bifurcation point at the time of specification, resulting in two distinct populations (Fig. 2*H* and *SI Appendix*, Fig. S2 *A* and *B*; Branched expression analysis modeling included in Dataset S13). We also noticed that a subset of E8.25 premigratory neural crest grouped together with the E7.5 anterior ectoderm within the most undifferentiated cell state to the left of the branch point, and we found that these cells were enriched for genes involved in RNA processing (Dataset S14). To determine the spatial distribution of each population of E8.25 premigratory neural crest, we examined the expression of medial/lateral markers in neuronal progenitors and ectomesenchyme (Fig. 2*H*). Early in the differentiation trajectory, the two populations were indistinguishable from one another including similar expression of lateral markers such as *E-cadherin* and *Krt8/19*. In contrast to ectomesenchyme, neuronal progenitors gained expression of medial markers at the end of the trajectory (*Sox1* and *N-cadherin*), consistent with later differentiation (Fig. 2*H*). To further investigate the order of emergence of each premigratory neural crest lineage, we analyzed their progression along a pseudotime trajectory. Ectomesenchyme cells had progressed further compared with neuronal progenitors (Fig. 2*I*), consistent with an abundance of ectomesenchyme previously reported at this time point (7). To further support this finding, we analyzed spatiotemporal formation at the neural plate border in mouse embryos using coimmunofluorescence staining for PAX3 (a pan-neural crest transcription factor) and either E-CADHERIN (ectomesenchymal) or SOX1 (neuronal lineage). We found that premigratory neural crest form within a neural plate border domain of the ectoderm that stains positive for both E-CADHERIN and SOX1 at E8.0 (Fig. 2*J* and *SI Appendix*, Fig. S2*D*). Specifically, SOX1 expression is up-regulated in medial cells of the neural plate, and weaker expression was observed in lateral cells at the neural plate border expressing PAX3 (Fig. 2*J* and *SI Appendix*, Fig. S2*D*). Conversely, E-CADHERIN is ubiquitously expressed throughout the ectoderm at E8.0 before medial downregulation, as expression is restricted to lateral PAX3-positive cells at the neural plate border between E8.25 and E8.5 (Fig. 2*J* and *SI Appendix*, Fig. S2*D*). As the SOX1-positive and E-CADHERIN-positive domains of the ectoderm refine and become mutually exclusive between E8.25 and E8.5, PAX3+ cells at the neural plate border begin to express either SOX1 or E-CADHERIN in a mutually exclusive pattern. In contrast to E8.0, PAX3 expression is enriched within SOX1-positive/E-CADHERIN-negative cells of the ectoderm at E8.25 and E8.5. (Fig. 2 *J* and *K* and *SI Appendix*, Fig. S2*D* and Dataset S15; wide field view of cross-sections provided in *SI Appendix*, Fig. S2*D*). These findings support a divergence of premigratory neural crest lineages as epithelial compartments of the ectoderm are being refined at the neural plate border. These data are consistent with previous mammalian and avian studies suggesting a general lateral to medial transition of neural plate border differentiation and that this movement can bias terminal fate (7).

To identify transcriptional regulators of premigratory neural crest specification, we mapped the expression of transcription factors enriched within E7.5 epiblast cells across both neuronal progenitors and ectomesenchyme. We found *Oct4*, *Nanog*, *Sox2,* and *Sall4* were expressed in both the E7.5 anterior ectoderm and distinct subsets of E8.25 premigratory cranial neural crest cells (Fig. 2*L* and *SI Appendix*, Fig. S2*C*). Interestingly, we observed differential expression of factors that are associated with early fate specification across the two populations of premigratory neural crest. For example, *Oct4* is preferentially expressed in the ectomesenchyme lineage along with related factors, such as *Oct2* (Fig. 2*L*). Whereas neuronal-biased cells preferentially expressed *Sall4*, *Otx2*, *Oct6,* and *Sox2* (Fig. 2*L*). Both populations expressed canonical neural crest transcription factors such as *Pax3* and *Sox9* (Fig. 2*L*). These data identify segregation of premigratory neural crest at the neural plate border and transcription factors that are differentially expressed in a lineage-specific manner within cranial neural crest cells. Our findings highlight spatiotemporal differences in neural crest specification, suggesting that neural crest initially form within a common domain at the neural plate border and are soon thereafter segregated into neural and nonneural domains of the ectoderm.

### Epiblast and Neural Crest Transcription Factors Form a Regulatory Network.

**Fig. 3.**
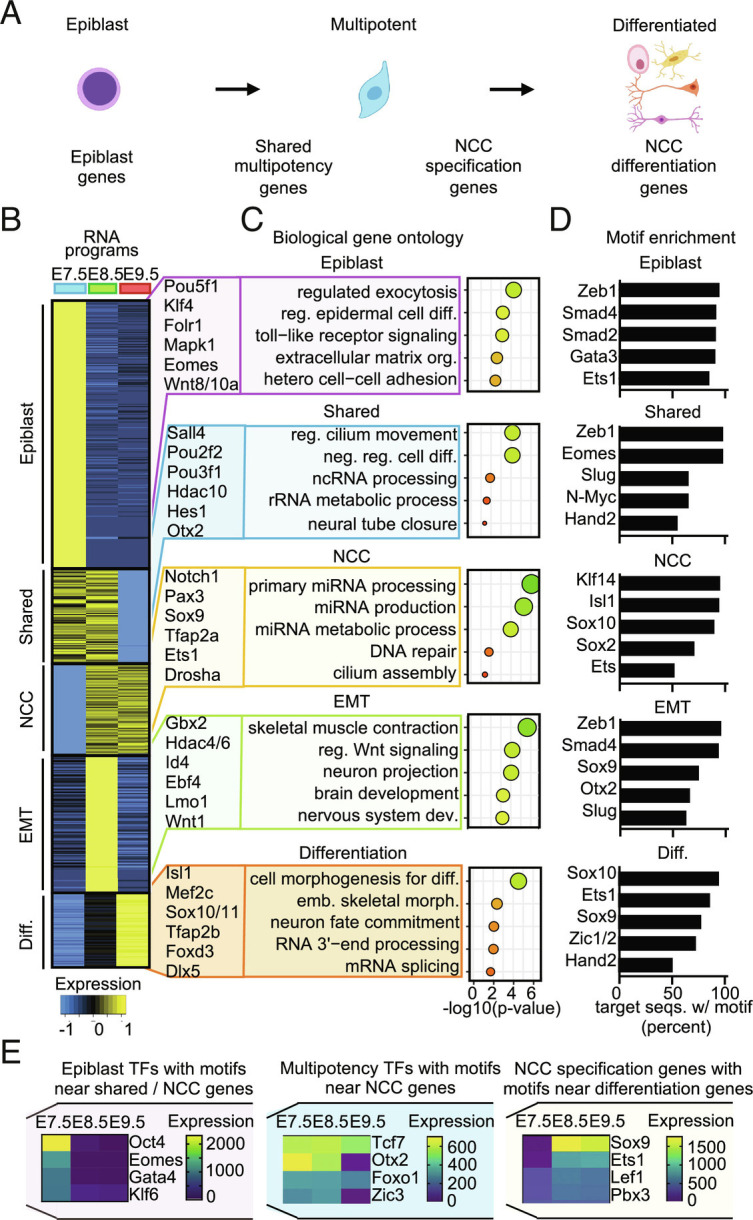
Epiblast and neural crest transcription factors form a regulatory network. (*A*) Schematic of differentiation potential and gene expression during neural crest development. (*B*) Clustering of gene expression changes from E7.5 epiblast and sorted neural crest cells at E8.5 (*Wnt1*-positive) and E9.5 (*Sox10*-positive). (*C*) Gene ontology analysis and (*D*) motif enrichment for each neural crest-associated gene cluster. Gene clusters, gene ontology, and enriched motifs are included in Dataset S16. (*E*) Comparison of motif enrichment and gene modules reveals factors expressed early on, which are predicted to regulate genes expressed at later stages of differentiation. Cluster and motif comparisons are included in Dataset S17.

To explore how genetic regulatory networks associated with the E7.5 epiblast are integrated into the neural crest gene regulatory network, we used RNA sequencing (RNA-seq) of the epiblast at E7.5 and sorted neural crest populations at E8.5 and E9.5 (*Wnt1*-positive cells at E8.5, and *Sox10*-positive cells at E9.5). We applied unsupervised clustering to define gene expression modules, used biological gene ontology analysis to predict function, and identified significantly enriched motifs in the promoter region of genes within each module. We examined modules related to neural crest development in more detail: epiblast, shared multipotency, neural crest specification, EMT, and differentiation (Fig. 3 *A* and *B* and *SI Appendix*, Fig. S3*A*; full dataset provided in Dataset S16). The epiblast module consisted of genes robustly expressed at E7.5 that declined and were minimally expressed at E8.5, such as *Oct4, Eomes,* and *Klf4,* which are involved in germ layer specification and differentiation (Fig. 3*B*). The shared multipotency module included genes that were expressed at both E7.5 and E8.5, but not at E9.5, such as *Oct2/6*, *Sall4,* and *Otx2*. The genes within this module were predicted to negatively regulate cell differentiation and be involved in the processing of noncoding RNAs (Fig. 3*C*). As expected, the neural crest specification module included genes such as *Pax3*, *Ets1*, *Sox9*, and *Tfap2a* that were expressed at E8.5 and E9.5. Interestingly, genes enriched in the neural crest specification module were predicted to function in miRNA processing (Fig. 3 *B* and *C*), similar to our analysis of scATAC-seq data (*SI Appendix*, Fig. S1*E*). Genes within the EMT module were robustly expressed at E8.5, but not E7.5 or E9.5 and were predicted to function in patterning, migration, and nervous system development (Fig. 3 *B* and *C*). Lastly, genes within the differentiation gene module increased in expression from E7.5 to E9.5 and included genes highly associated with migratory identity such as *Sox10, Tfap2b, and FoxD3* (6, 36–38) (Fig. 3 *B* and *C*).

To further uncover predicted gene regulatory networks associated with each module, we identified significantly enriched transcription factor-binding motifs in the promoter region of genes within each module. We noticed that transcription factors expressed early during development were predicted to bind regulatory elements of genes expressed at later stages (Fig. 3 *D* and *E*). For example, *Eomes* was expressed at E7.5 and had significantly enriched motifs in genes that were expressed at both E7.5 and E8.5 (Fig. 3 *B*–*E*). By comparing the genes and motifs within each cluster, we observed a model that predicts a sequential handoff in transcriptional control (full dataset provided in Dataset S1). Earlier factors were predicted to bind to the promoter region of genes expressed at later stages of differentiation, similar to the sequential avian neural crest gene regulatory network (35–38). In summary, our results suggest that transcription factors from the epiblast are critical upstream regulators of the mammalian neural crest gene regulatory network. Further work in mammalian neural crest cells will be needed to demonstrate the direct binding of epiblast transcription factors to regulate neural crest genes.

### miR-302 Is Expressed in Cranial Neural Crest Cells.

**Fig. 4.**
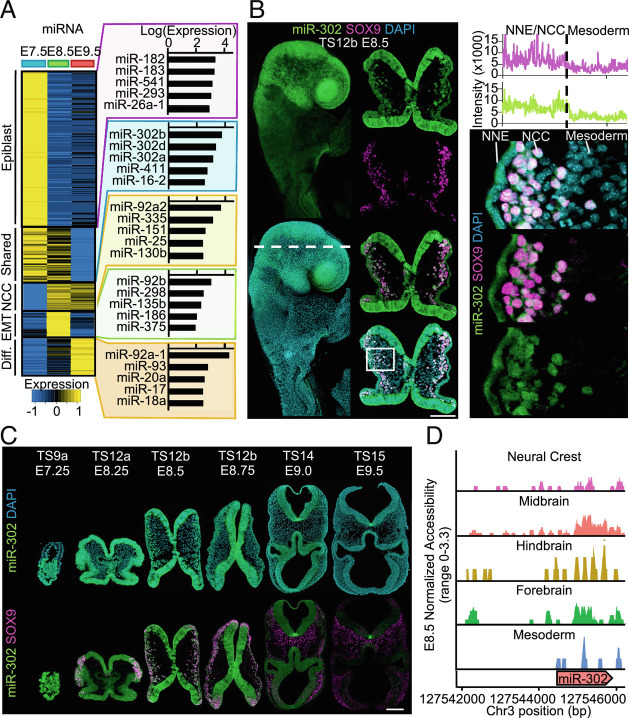
miR-302 is expressed in cranial neural crest cells. (*A*) Clustering of miRNA expression changes from E7.5 epiblast and sorted neural crest cells at E8.5 and E9.5. MiR-302 is the most highly expressed miRNA maintained from E7.5 to E8.5 neural crest cells. MiRNA clustering analysis included in Dataset S18. (*B*) Wholemount miR-302-eGFP embryos (TS12b, 6 somites) and transverse cross-sections through the cranial region (TS12b, 5 somites). Immunofluorescence for SOX9 reveals colocalization with miR-302-eGFP. Signal intensity of miR-302-eGFP reveals miR-302 expression in neural crest (NCC) and nonneural ectoderm (NNE), while minimal signal was observed in cranial mesoderm. (Scale bar, 100 μm.) (*C*) Immunofluorescence for GFP and SOX9 in sagittal cross-section at E7.25 (TS9a, 0 somites) and transverse cross-section at E8.25 (TS12a, 3 somites), E8.5 (TS12b, 5 somites), E8.75 (TS12b, 7 somites), E9.0 (TS14, 15 somites), and E9.5 (TS15, 21.5 somites). At three to seven somites of age, miR-302-eGFP colocalizes with SOX9. (Scale bar, 100 μm.) (*D*) Coverage plots of accessibility at the *mir-302* locus in E8.5 ectoderm-derived cell populations and minimal chromatin accessibility in the cranial mesoderm.

Since we found that both increased chromatin accessibility and gene expression coincides with enrichment of the miRNA regulatory pathway, we hypothesized that miRNAs may have an important role in cranial neural crest specification. To identify candidate miRNAs, we performed small RNA-seq on the same progenitor and neural crest populations from our bulk RNA-seq experiment (E7.5 epiblast, E8.5 *Wnt1*-positive cells, and E9.5 *Sox10*-positive neural crest). At E7.5, miR-302 was the most highly expressed miRNA in the epiblast. Supporting an important role in pluripotent cells, miR-302 is robustly expressed in human embryonic stem cells and sufficient to convert somatic cells into induced pluripotent stem cells (22–24). At E8.5, miR-302 was the second-most highly expressed miRNA in premigratory and migratory neural crest cells, second only to *mir-92* (*SI Appendix*, Fig. S4*A*). Deletion of miR-92 results in cleft palate and bone deficiencies (39, 40), and we find that *mir-92* expression increases during neural crest development from E7.5 to E9.5 suggesting a role in differentiation of neural crest derivatives, rather than neural crest specification or maintaining potential of neural crest cells. Robust expression of miR-302 was maintained and did not change significantly from E7.5 to E8.5, but its expression significantly declined at E9.5 (*SI Appendix*, Fig. S4*B*). To identify shared miRNAs in pluripotency and cranial neural crest, we defined miRNA modules using unbiased clustering and obtained a total of seven clusters. We further explored five clusters corresponding to the previously defined gene modules: pluripotency, shared multipotency, neural crest specification, EMT, and differentiation (Fig. 4*A*; Full clustering analysis provided in Dataset S18). Indeed, the miR-302 family members were the most highly expressed miRNAs in the shared multipotency module representing miRNAs highly expressed in both E7.5 epiblast and E8.5 cranial neural crest cells (Fig. 4*A*). For reference, miR-302 was expressed at similar or higher levels than miRNAs previously associated with neural crest development, such as miR-140, which regulates palatogenesis in zebrafish neural crest development (41) (*SI Appendix*, Fig. S4*C*). While miRNAs associated with primed pluripotency, such as miR-302, were among the most highly expressed miRNAs at E7.5 and E8.5, miRNAs associated with naïve pluripotency, such as miR-293 (part of the naïve-specific miR-290 ~ 295 cluster), were expressed exclusively at E7.5 (*SI Appendix*, Fig. S4*D*). To validate the expression of miR-302, we used an established *mir-302-eGFP* reporter mouse (42) and analyzed expression at E7.25 to E9.5 (*SI Appendix*, Fig. S4*E*). MiR-302-eGFP was enriched within the cranial region compared with the trunk (Fig. 4*B* and *SI Appendix*, Fig. S4*E*). At E7.5, MiR-302-eGFP was expressed in the epiblast and at E8.5 MiR-302-eGFP signal colocalized with both premigratory and migratory neural crest (Fig. 4 *B* and *C*). Importantly, miR-302-eGFP was expressed at E8.25 in ectoderm as neural crest are specified (Fig. 4 *B* and *C*). MiR-302-eGFP declines in neural crest cells starting at E9.0 and is largely lost by E9.5 (Fig. 4*C*).

To determine how miR-302 expression is maintained from the epiblast during cranial neural crest development, we analyzed our single-cell ATAC-seq data to interrogate chromatin accessibility around the *mir-302* locus. Consistent with distinct regulatory mechanisms, regions of accessibility were different upstream of the *mir-302* locus across cell populations that express miR-302 including the neural crest, neural tube, and nonneural ectoderm. Regions of accessible chromatin upstream of the miR-302 locus were minimal in cell populations that do not express miR-302 such as the cranial mesoderm (Fig. 4*D*). Together, these data suggest distinct mechanisms of transcriptional regulation controlling miR-302 expression across postgastrulation lineages.

### mir-302 Differentially Regulates Specification of Ectomesenchymal versus Neuronal Progenitor Neural Crest.

**Fig. 5.**
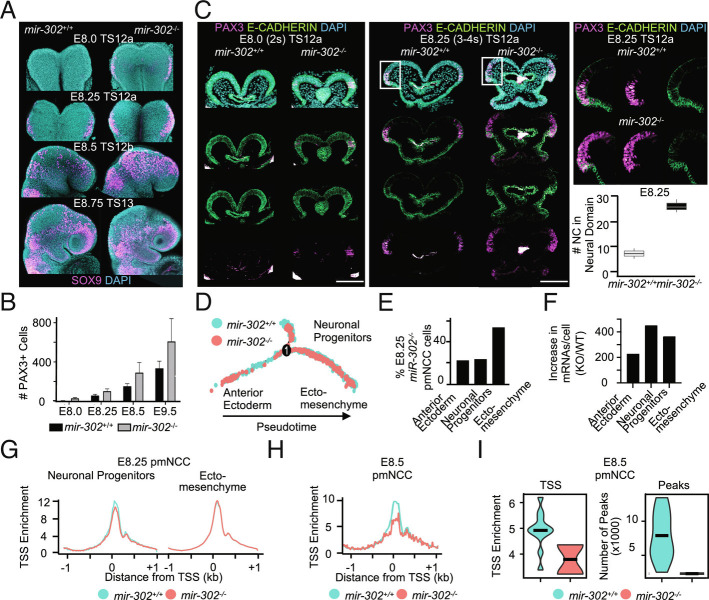
*mir-302* differentially regulates specification of ectomesenchymal versus neuronal progenitor neural crest. (*A*) Wholemount immunofluorescence for SOX9 in *mir-302^+/+^* and *mir-302^−/−^* embryos at E8.0 (2 somites, TS12a), E8.25 (4 somites, TS12a), E8.5 (7 somites TS12b), and E8.75 (9 somites, TS13). (*B*) Boxplot of PAX3-positive cells over time. ANOVA *mir-302^+/+^* versus *mir-302^−/−^* E8.0 *P* = 0.015, E8.25 *P* = 0.038, E8.5 *P* = 0.049, E9.5 *P* = 0.094. Raw data available in Dataset S19. (*C*) Immunofluorescence of *mir-302^+/+^* and *mir-302^−/−^* transverse cranial cross-sections for E-CADHERIN and PAX3 at E8.0 (2 somites, TS12a) and E8.25 (3 to 4 somites, TS12a). (Scale bar, 50 μm.) Zoomed-in view of the *mir-302^+/+^* and *mir-302^−/−^* neural plate border. Quantification of the number of PAX3-positive cells in the E-CADHERIN-negative domain of *mir-302^+/+^* and *mir-302^−/−^* ectoderm at E8.25 revealed a significant expansion of the neural plate border into the neural domain upon *mir-302* deletion (ANOVA *P* = 0.00059). Raw data available in Dataset S20.(*D*) Pseudotime trajectory comparing the *mir-302^+/+^* and *mir-302^−/−^* E7.5 anterior ectoderm and E8.25 cranial neural crest. (*E*) Quantification of cell distribution and (*F*) total mRNA increase, in each cell state of the *mir-302^−/^^−^* relative to *mir-302^+/+^*. Raw data available in Dataset S21. (*G*) TSS enrichment of the *mir-302^+/+^* and *mir-302^−/−^* ectomesenchyme and neuronal progenitor lineages of neural crest. A slight reduction in TSS accessibility is observed in the neuronal progenitor lineage. Raw data available in Dataset S22. (*H*) TSS enrichment in E8.5 *mir-302^+/+^* and *mir-302^−/−^* premigratory neural crest cells. Raw data available in Dataset S22. (*I*) Violin plots comparing the TSS enrichment and the number peaks in E8.5 *mir-302^+/+^* and *mir-302^−/−^* premigratory neural crest cells.

To determine how miR-302 regulates cranial neural crest development, we used CRISPR/Cas9 genome editing to genetically delete miR-302 and compared neural crest development between *mir-302^−/−^* and *mir-302^+/+^* embryos from E8.0 to E9.5 (Fig. 5 *A* and *B* and *SI Appendix*, Fig. S5 *A*–*F*; locus details and strain validation are provided within *SI Appendix*, Fig. S5 *A*–*C*; strain generation information is provided within the *SI Appendix*). Sanger sequencing and genotyping identified the deletion allele and bulk small RNA sequencing of neural crest cells confirmed the loss of *mir-302* expression (*SI Appendix*, Fig. S5 *A* and *C*). Bulk mRNA sequencing of neural crest cells revealed no significant change in expression of the *mir-302* host gene, *Larp7* (*SI Appendix*, Fig. S5*B*). Consistent with previous findings, we observed a cranial neural tube closure defect upon *mir-302* deletion (*SI Appendix*, Fig. S5*E*) (42). Since miR-302 is expressed in both anterior ectoderm and neural crest cells (Fig. 4), maintains embryonic stem cell self-renewal (22–24), and regulates developmental timing of differentiation within the neural tube (42), we first asked whether miR-302 prevents precocious specification of cranial neural crest. Indeed, loss of *mir-302* resulted in precocious expression of SOX9 at the neural plate border at E8.0 and a global increase in the number of neural crest cells in *mir-302^−/−^* embryos from E8.0 to E9.5 (Fig. 5 *A* and *B* and *SI Appendix*, Fig. S5 *D*–*F*, and Dataset S19).

To determine whether *mir-302* deletion affects ectomesenchyme and/or neuronal premigratory neural crest, we performed coimmunofluorescence for PAX3 and E-CADHERIN and analyzed neural plate border localization in *mir-302^−/−^* embryos. Loss of *mir-302* at E8.0 resulted in precocious expression of PAX3 in the lateral E-CADHERIN-positive domain (i.e., ectomesenchyme) (Fig. 5*C*). Later, at E8.25, we found that *miR-302* deletion resulted in a significant medial expansion of the neural plate border into the neural domain of the ectoderm, thus both lineages (i.e., ectomesenchyme and neuronal progenitors) appear to develop precociously in *mir-302^−/−^* embryos (Fig. 5*C* and Figure 5 – Dataset S20). To identify changes in gene expression and chromatin accessibility of premigratory neural crest upon *mir-302* deletion, we used single-cell mRNA and ATAC sequencing of *mir-302^−/−^* embryos at E8.25 and integrated the data with a somite-matched *mir-302^+/+^*embryo. Cells of the E7.5 anterior ectoderm and E8.25 premigratory neural crest were aligned along a pseudotime trajectory revealing that the majority of *mir-302^−/−^* neural crest cells were part of the ectomesenchyme lineage (Fig. 5 *D* and *E* and Dataset S21). However, both ectomesenchyme and neuronal-progenitor lineages of premigratory neural crest had an increase in the number of mRNAs per cell, suggesting miR-302 negatively regulates transcript stability in both populations (Fig. 5*F* and Dataset S21). Analysis of TSS accessibility revealed minimal change in the ectomesenchyme lineage in contrast to a small decrease in neuronal progenitors (Fig. 5*G* and Dataset S22).

Next, to focus on the formation of neuronal progenitors, we compared single-cell mRNA and ATAC sequencing of *mir-302^−/−^* embryos at E8.5 that were stage-matched to *mir-302^+/+^* embryos with identical somite counts. Interestingly, we identified a significant reduction of chromatin accessibility in E8.5 premigratory cranial neural crest following the loss of *mir-302,* the timepoint when a significant number of neural crest emerge from the neural domain (Figs. 2*K* and 5 *H* and *I* and Dataset S22). Minimal changes in chromatin accessibility are observed in *mir-302^−/−^* neural crest at E7.5 and E8.25 (*SI Appendix*, Fig. S5*G*). These results suggest that at the time when significantly more neural crest are forming as a part of the neuronal domain, miR-302 may be critical for the differentiation capacity of this lineage of neural crest. Taken together, our findings are consistent with a role for *miR-302* to control the timing of neural crest specification and chromatin accessibility. Together, our results demonstrate that posttranscriptional regulation by miR-302 is required during cranial neural crest development.

### miR-302 Targets Sox9 to Restrain Specification of the Ectomesenchyme Lineage.

**Fig. 6.**
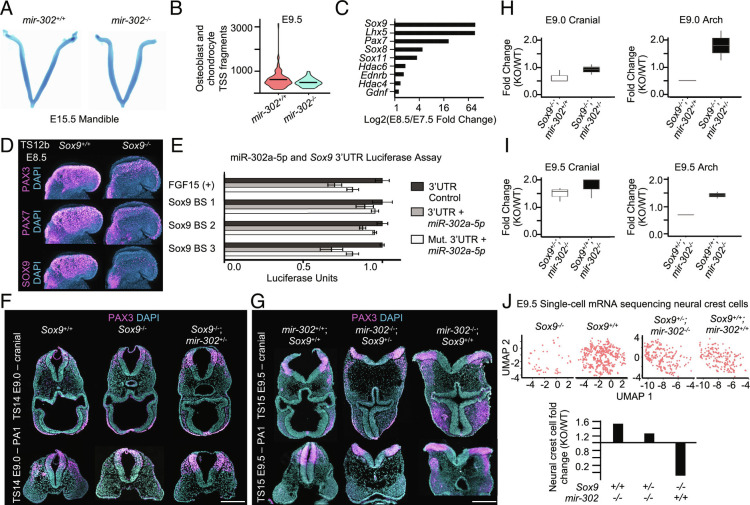
miR-302 targets *Sox9* to restrain specification of the ectomesenchyme lineage. (*A*) Alcian blue staining of E15.5 *mir-302^+/+^* and *mir-302^−/−^* mandibles. (*B*) Violin plot of TSS fragments showing no change in accessibility of the E9.5 ectomesenchyme lineage. (*C*) Change in expression from E7.5 to E8.5 of up-regulated miR-302 targets that are known neural crest genes. (*D*) Wholemount immunofluorescence of E8.5 (TS12b, 5 somites) *Sox9^+/+^* and *Sox9^−/−^* embryos for PAX3, PAX7, and SOX9. (*E*) Luciferase assay to demonstrate the direct binding of miR-302 to the 3′UTR of *Sox9* (ANOVA for binding sites 2 and 3 and Fgf15-positive control *P* < 0.05). The *miR-302a* 5p seed sequence (TTAAAG) was mutated to (TTGACG) within each seed sequence. Plasmids were transfected into human embryonic stem cells and normalized using internal control luciferase. Error bars represent SD. Raw data available in Dataset S24. (*F*) Transverse cross-sections through the cranial region and pharyngeal arch 1 of E9.0 (TS14) *Sox9^+/+^*; *miR-302^+/+^, Sox9^−/−^*, and *Sox9^−/−^*; and *miR-302^+/−^* embryos. (Scale bar, 100 μm.) (*G*) Quantification of the fold change of the number of PAX3-positive cells from the cranial and pharyngeal arch sections of E9.0 (TS14) *Sox9^+/+^*; *miR-302^+/+^, Sox9^−/−^*, and *Sox9^−/−^*; and *miR-302^+/−^* embryos. Raw data available in Dataset S25. (*H*) Transverse cross-sections through the cranial region and pharyngeal arch of E9.5 (TS15) *Sox9^+/+^*; *miR-302^+/+^, miR-302^−/−^*, and *miR-302^−/−^*; *Sox9^+/−^* embryos. (Scale bar, 100 μm.) (*I*) Quantification of the fold change of the number of PAX3-positive cells from the cranial and pharyngeal arch sections of E9.5 (TS15) *Sox9^+/+^*; *miR-302^+/+^, miR-302^−/−^*, and *miR-302^−/−^*; *Sox9^+/−^* embryos. Raw data available in Dataset S25. (*J*) UMAP plots and quantification of neural crest cell number of *miR-302^−/−^* and *miR-302^−/−^; Sox9^+/−^* embryos compared with *Sox9^+/+^*; *miR-302^+/+^*. Raw data available in Dataset S26.

To determine if miR-302 is required for proper development of the ectomesenchyme lineage, we analyzed terminal differentiation into chondrocytes using Alcian blue staining, which revealed minimal changes after deletion of *mir-302* (Fig. 6*A* and *SI Appendix*, Fig. S6 *A* and *B*). scRNA-seq of the cranial region at E9.5 revealed an increase in expression of genes that promote the chondrocyte fate in E9.5 migratory neural crest cells, as well as an increase in the number of osteoblast precursors (*SI Appendix*, Fig. S6 *C* and *D*). Analysis of scATAC-seq data at E9.5 showed minimal changes in accessibility of the chondrocyte lineage, thus miR-302 is not required for terminal differentiation of chondrocytes (Fig. 6*B* and *SI Appendix*, Fig. S6*E*). To further understand how miR-302 prevents precocious specification of ectoderm into ectomesenchymal neural crest, we focused on up-regulated miR-302 targets at E7.5. We compared a list of genes previously associated with neural crest formation to up-regulated targets in the *mir-302^−/−^* at our earliest time point of E7.5. Consistent with our results, we found that miR-302 is predicted to target many well-studied neural crest factors. Of these, *Sox9* had the most dramatic increase in expression during formation (Fig. 6*C*). Although Sox9 has been thoroughly investigated in nonmammalian neural crest specification (2, 29, 43) and mammalian chondrocyte differentiation (44), its role in early specification of cranial neural crest in mice has not been carefully examined. Therefore, we used a neural crest-specific conditional deletion of *Sox9* by crossing the *Sox9^ f/f^*mouse (45) with Wnt1-Cre (46) Interestingly, we found a significant reduction of neural crest upon *Sox9* deletionat E8.5 (Fig. 6*D* and *SI Appendix*, Fig. S6*F* and Dataset S23). To validate the direct targeting of *Sox9* by *mir-302*, we used a luciferase assay in which we transfected 293T cells with plasmids containing wild type and mutated miR-302a-5p-binding sites in the 3’UTR of *Sox9*. Using miR-302a-5p mimics, we found evidence of direct binding of miR-302a-5p in the 3′UTR of *Sox9,* which is disrupted upon mutation of the seed sequence (Fig. 6*E* and Dataset S24). To determine whether posttranscriptional regulation of Sox9 controls the timing of ectomesenchyme specification, we first asked whether deletion of one copy of miR-302 could rescue the reduction of neural crest formation of the Sox9^−/−^ embryo, thus we compared neural crest in Sox9^−/−^; *mir-302^+/^*^−^ embryos to Sox9^−/−^ embryos and found an increase in PAX3-positive neural crest cells. To further interrogate the posttranscriptional regulatory axis of mir-302/Sox9, we deleted one copy of Sox9 in mir-302^−/−^ embryos and observed a decrease in the number of PAX3-positive cells compared with mir-302^−/−^ embryos (Fig. 6 *F-I* and *SI Appendix*, Fig. S6 *G-I* and Dataset S25). To further validate this finding, we used single-cell sequencing of a *mir-302^−/−^; Sox9^−/−^*, and *mir-302^−/−^*; *Sox9^+/−^* embryo and compared the number of neural crest with a *mir-302^+/+^; Sox9^+/+^* embryo at E9.5 (Fig. 6*J*). In the *mir-302^−/−^* embryo, we observed a 1.5-fold increase in neural crest specification, whereas in the *mir-302^−/−^*; *Sox9^+/−^* embryo, this was reduced to 1.3-fold (Fig. 6*J* and Dataset S26). Our demonstration of partial genetic rescue in *mir-302^−/−^*; *Sox9^+/−^* embryos establishes a critical role for posttranscriptional regulation of *Sox9* to control specification of the ectomesenchymal lineage of neural crest.

### miR-302 Is Required for Differentiation of the Neuronal Lineage of Cranial Neural Crest.

**Fig. 7.**
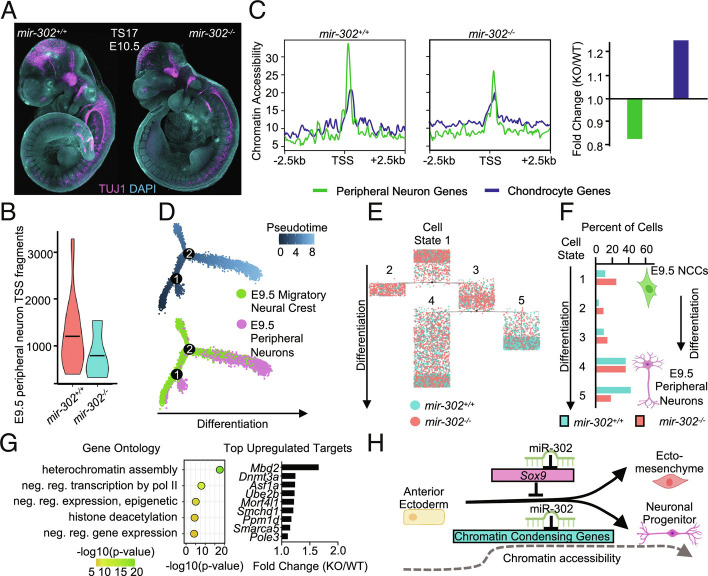
miR-302 is required for differentiation of the peripheral neuron lineage of cranial neural crest. (*A*) Wholemount immunofluorescence for TUJ1 in *mir-302^+/+^* and *mir-302^−/−^* embryos at E10.5 (TS17). (*B*) Violin plot of TSS fragments showing a reduction in accessibility of the E9.5 peripheral neuron lineage. (*C*) Peak plot and bar plot comparing chromatin accessibility of peripheral neuron and chondrocyte genes. (*D*) To assess whether *mir-302* deletion results in a block in peripheral neuron differentiation, E9.5 neural crest cells and E9.5 peripheral neurons were aligned along a pseudotime trajectory. Differentiation trajectory of E9.5 neural crest cells and E9.5 peripheral neurons reveals two branch points, resulting in five cell states. (*E*) Plot showing cells residing in each cell state during differentiation of E9.5 neural crest into peripheral neurons, colored by genotype. (*F*) Percent of *mir-302^+/+^* and *mir-302^−/−^* cells residing in each cell state. E9.5 peripheral neurons of the *mir-302^−/−^* embryo reside in the most undifferentiated cell state. Raw data available in Dataset S28. (*G*) Gene ontology of miR-302 targets that regulate chromatin accessibility predicts involvement in heterochromatin assembly and histone deacetylation consistent with a reduction in chromatin accessibility upon *mir-302* deletion. Top up-regulated miR-302 targets in *mir-302^−/−^* E8.5 premigratory neural crest that regulate chromatin accessibility, many of which are known to methylate histones. Raw data available in Dataset S29. (*H*) Schematic demonstrating the maintenance of developmental potential of the ectoderm during formation of the ectomesenchyme lineage by miR-302 via direct targeting of *Sox9*, and the maintenance of developmental potential of the neuronal progenitor lineage by miR-302 via predicted targeting of chromatin condensing genes.

Since we found reduced chromatin accessibility in premigratory neural crest at E8.5, we next asked whether miR-302 controls the ability of neural crest to differentiate into derivatives of the neuronal lineage. Indeed, we find a reduction of peripheral neurons in *mir-302^−/−^* embryos compared with *mir-302^+/+^* at E10.5 (Fig. 7*A*). To determine whether impaired neuronal differentiation was a cell-autonomous defect, we used a neural tube explant assay to isolate migratory neural crest cells to assess neuronal differentiation (47, 48) (*SI Appendix*, Fig. S7 *A*–*D*). Similar to our in vivo observations, we found a 10-fold reduction in neuronal differentiation of *mir-302^−/−^* neural crest cells ex vivo (*SI Appendix*, Fig. S7 *C* and *D*; and Dataset S27). To determine whether miR-302 regulates chromatin accessibility, we used scATAC-seq at E8.5 to examine specification of neuronal-progenitors. Consistent with a defect in neuronal differentiation, we found that TSS accessibility was reduced in E9.5 peripheral neurons upon miR-302 deletion (Fig. 7*B*). Indeed, we found that chromatin accessibility around peripheral neuron genes decreased 0.8-fold in *mir-302^−/−^* embryos compared with *mir-302^+/+^*, whereas accessibility around chondrocyte genes was increased 1.2-fold (Fig. 7*C* and *SI Appendix*, Fig. S7*E*). To better understand the increased number of neural crest and associated decrease of neuronal differentiation observed in *mir-302^−/−^* embryos, we used single-cell RNA-seq at E9.5 and aligned migratory neural crest cells along a pseudotime trajectory (Fig. 7*D*). *Mir-302^−/−^* cells accumulated at early steps in the differentiation trajectory (20% in *mir-302^−/−^* vs. 10% in *mir-302^+/+^* in cell state 1), leading to a decrease in differentiated cell states of the neuronal lineage (<25% in *mir-302^−/−^* vs. 40% in *mir-302^+/+^* in cell state 5; Fig. 7 *E* and *F* and Dataset S28). These data suggest that miR-302 promotes increased accessibility of chromatin in neuronal-progenitors during specification, which is required for terminal differentiation of peripheral neurons. Together, these data suggest that miR-302 has a distinct role in maintaining chromatin accessibility required for differentiation of the neuronal progenitor neural crest.

To better understand how miR-302 controls accessibility of chromatin, we further analyzed our scRNA-seq data at E8.25 to identify misregulated transcripts that could be leading to a defect at E8.5. Thus, we identified up-regulated miR-302 targets in *mir-302^−/−^* embryos at E8.25 and queried their predicted function in regulating chromatin organization using gene ontology analysis. Consistent with our observation of reduced chromatin accessibility, we found that up-regulated targets of miR-302 were predicted to be involved in heterochromatin assembly, histone deacetylation, and negative regulation of transcription (Fig. 7*G* and Dataset S29). Top up-regulated predicted miR-302 targets that regulate chromatin accessibility included *Mbd2* and *Dnmt3a* (Fig. 7*G* and Dataset S29). These findings suggest the role of miR-302 is context dependent across time and distinct lineages of cranial neural crest. MiR-302 directly targets *Sox9* to control the timing of ectomesenchymal specification and is predicted to target chromatin condensing genes to maintain chromatin accessibility required for differentiation of peripheral neurons.

## Discussion

To what degree early cranial neural crest reflect a population of homogeneous multipotent stem cells versus heterogeneous population of progenitors has long been of interest for regenerative medicine, vertebrate evolution, and as a therapeutic target for craniofacial birth defects (1–15, 29, 43, 49, 50). Mammalian models have faced challenges due to a lack of available tools to study neural crest specification (51). Our study attempts to overcome this limitation using multiple approaches to carefully profile how chromatin accessibility, miRNA, and gene expression change during neural crest specification. Our results, together with previous findings, suggest increased chromatin accessibility (18), miRNAs, and transcription factors (6, 11) are shared between somatic cell reprograming and cranial neural crest specification. Furthermore, our single-cell multiomic approach allowed for the characterization of two distinct populations of premigratory cranial neural crest cells that were similarly identified by other groups (5, 7). We find that each population is enriched for different lineage-specific genes, differentially accessible peaks, and transcription factor motifs, in line with previous results (52, 53). Our findings suggest that the heterogeneity of cells observed at the neural plate border reflects differential fate bias of ectomesenchyme and neuronal progenitors. The increase in chromatin accessibility from TS11d at E7.5 to TS12a at E8.25 may allow for simultaneous lineage fate bias, subsequent activation of migratory programs and expression of factors from earlier stages of development. Careful profiling of chromatin accessibility during E7.5 through the progression of TS11a-c before cranial neural crest progenitors can be identified from the ectoderm will help to further delineate accessibility changes during neural crest specification. Recent single-cell studies of cranial neural crest development have found that delaminating neural crest share similar transcriptional profiles suggesting previous positional information is erased (3, 6, 54). However, single-cell analysis of Wnt1-Cre+ cells sorted from four somite stage embryos also reveals multiple progenitor populations similar to our results (6). Indeed, Wnt1-Cre marked both Oct4 positive and negative neural crest precursors, suggesting that multiple distinct types of neural crest are specified shortly after gastrulation. The finding that Oct4 regulates ectomesenchyme (6) and miR-302 regulates neuronal differentiation (this study) further suggests that differential fate bias occurs at the earliest stages of cranial premigratory neural crest specification.

Our findings reveal unique factors of early development enriched in each population of neural crest and differential posttranscriptional regulation for proper fate specification and differentiation. For example, we and others have found enrichment of *Oct4* in the ectomesenchymal lineage (6), which is consistent with studies in embryonic stem cells that showed *Oct4* promotes mesendodermal and inhibits neural ectoderm fate during exit from pluripotency (55). Moreover, deletion of *Oct4* resulted in defects within the ectomesenchymal but not peripheral neuronal lineage in mice (6). However, *Oct4* was dispensable for the formation of neural crest (6), which suggests additional mechanisms control the timing of ectomesenchymal specification. In contrast to the ectomesenchyme lineage, the neuronal progenitor lineage repurposed different factors such as *Sall4*, *Sox2,* and *Otx2.* In a complementary, but opposite role of *Oct4*, *Sox2* inhibits mesendodermal fate and promotes neural ectoderm fate during germ layer specification of embryonic stem cells (55). Our results suggest that a majority of the neural plate border is specified to the neuronal progenitor population at E8.5. Interestingly, we also find reduced chromatin accessibility in E8.5 *mir-302^−/−^* neural crest and a defect in peripheral neuron differentiation, consistent with increased chromatin accessibility being required for neuronal differentiation. Indeed, mutations in chromatin remodelers have been identified as the cause of several craniofacial syndromes, illustrating the relevance of understanding chromatin dynamics during neural crest development (56–58).

The idea that chromatin structure could be modulated by miRNA control of chromatin remodelers is an attractive method to direct cell differentiation for cell fate engineering and regenerative medicine (59–67). Future studies are needed to better understand how miRNAs control fate specification by modulating the expression of genes that control chromatin accessibility in both pluripotent and neural crest cells and determining which chromatin remodelers control the dynamic changes observed during early neural crest development. Our observation of two distinct populations of premigratory cells that were differentially affected by loss of miR-302 provides an additional mechanistic explanation as to why some defects occur in cranial neural crest cartilage versus neuronal lineages in humans. Since the function of miR-302 is conserved in mice and humans (42) and *Sox9* deletion results in defects in cranial chondrocyte differentiation (44), we hypothesize that disruption of the miR-302 regulatory axis may underlie some human craniofacial and neurological disorders. Consistent with our findings, Dicer was reported to be up-regulated in chicken neural crest cells (68). Thus, precocious differentiation and early expansion of neural crest following deletion of miR-302 suggests that posttranscriptional regulation is a conserved mechanism of regulation during cranial neural crest specification. In the present study, our approach to studying the lineage-specific role of miR-302 in neural crest cells lacks cellular specificity, as there is currently no method of genetically deleting or lineage tracing subsets of premigratory neural crest cells independently. Future studies will be critical for addressing outstanding questions regarding the lineage-specific role of miR-302. For example, we find that *Sox9* is expressed comparably within both lineages of neural crest (Fig. 2*B*) and with our current approach to dissect the role of the *mir-302/Sox9* axis in ectomesenchymal neural crest, it remains to be determined whether *mir-302* could be targeting *Sox9* in the neural lineage of premigratory neural crest to inhibit ectomesenchymal fate. Lineage-specific tools will also be critical for determining subtle differences in timing of formation, migration, and terminal differentiation of each population of premigratory neural crest. For example, as neural crest form during development, they are exposed to different environmental cues suggesting that early delaminating leader cells have similar potential to later delaminating cells that follow (69). Determining the premigratory population that is the first to undergo EMT, then lineage tracing these cells and determining their terminal fate will be important for delineating differences between early leader versus follower populations. MiR-302 deletion results in precocious specification of ectomesenchyme at E8.0 and an increase in both the number of osteoblast precursors and in expression of chondrocyte genes at E9.5. However, Alcian blue staining revealed that chondrocyte derivatives of *mir-302^−/−^* embryos appear similar to wild type at E15.5. It remains unknown whether the *mir-302^−/−^* ectomesenchymal neural crest cells undergo apoptosis, or whether signaling and patterning processes within the pharyngeal arches can balance these transcriptional changes to regulate terminal differentiation and compensate for early differences in cell number (70–74).

Our results, together with previous studies, suggest that both transcription factors and miRNAs involved in primed pluripotency are critical regulators of the earliest cranial crest cells. Our findings begin to unravel the role of miR-302 during the transition from epiblast to neural crest and subsequent craniofacial development (16, 60–67). Since OCT4, NANOG, and SOX2 bind to the *mir-302* promoter region in pluripotent cells (66) and pluripotency is transiently lost following gastrulation, it will be interesting to determine how miR-302 is maintained (i.e., not reactivated) in neural crest cells. Similarly, the mechanisms driving reactivation of pluripotency programs remain unknown. A limitation of transcriptomic studies is a lack of information on protein levels. While we demonstrate a lineage-specific transcriptional reactivation of pluripotency transcription factors in neural crest cells, the translation of these factors has not been thoroughly investigated. In summary, our study reveals posttranscriptional regulation as a critical regulator of expanded developmental potential and timing of cranial neural crest development.

## Supplementary Material

Appendix 01 (PDF)

Dataset S01 (XLSX)

Dataset S02 (XLSX)

Dataset S03 (XLSX)

Dataset S04 (XLSX)

Dataset S05 (XLSX)

Dataset S06 (XLSX)

Dataset S07 (XLSX)

Dataset S08 (XLSX)

Dataset S09 (XLSX)

Dataset S10 (XLSX)

Dataset S11 (XLSX)

Dataset S12 (XLSX)

Dataset S13 (CSV)

Dataset S14 (XLSX)

Dataset S15 (XLSX)

Dataset S16 (XLSX)

Dataset S17 (XLSX)

Dataset S18 (XLSX)

Dataset S19 (XLSX)

Dataset S20 (XLSX)

Dataset S21 (XLSX)

Dataset S22 (XLSX)

Dataset S23 (XLSX)

Dataset S24 (XLSX)

Dataset S25 (XLSX)

Dataset S26 (XLSX)

Dataset S27 (XLSX)

Dataset S28 (XLSX)

Dataset S29 (XLSX)

## Data Availability

Raw and processed single-cell mRNA and single-cell ATAC data can be found under GEO SuperSeries GSE216138 and GSE216136. Bulk mRNA sequencing data can be found on the GEO database under GSE137227. All study data are included in the article and/or *SI Appendix*.

## References

[1] E. Theveneau, R. Mayor, Neural crest migration: Interplay between chemorepellents, chemoattractants, contact inhibition, epithelial-mesenchymal transition, and collective cell migration. Wiley Interdiscip. Rev. Dev. Biol. **1**, 435–445 (2012). 23801492 10.1002/wdev.28

[2] M. Simões-Costa, M. E. Bronner, Establishing neural crest identity: A gene regulatory recipe. Development **142**, 242–257 (2015). 25564621 10.1242/dev.105445 PMC4302844

[3] R. Soldatov , Spatiotemporal structure of cell fate decisions in murine neural crest. Science **364**, eaas9536 (2019). 31171666 10.1126/science.aas9536

[4] N. M. Le Douarin, S. Creuzet, G. Couly, E. Dupin, Neural crest cell plasticity and its limits. Development **131**, 4637–4650 (2004). 15358668 10.1242/dev.01350

[5] J. A. Weston , Neural crest and the origin of ectomesenchyme: Neural fold heterogeneity suggests an alternative hypothesis. Dev. Dyn. **229**, 118–130 (2004). 14699583 10.1002/dvdy.10478

[6] A. Zalc , Reactivation of the pluripotency program precedes formation of the cranial neural crest. Science **371**, eabb4776 (2021). 33542111 10.1126/science.abb4776 PMC8557957

[7] R. T. H. Lee , Cell delamination in the mesencephalic neural fold and its implication for the origin of ectomesenchyme. Development **140**, 4890–4902 (2013), 10.1242/dev.094680. 24198279 PMC4074292

[8] E. Buitrago-Delgado, K. Nordin, A. Rao, L. Geary, C. LaBonne, Shared regulatory programs suggest retention of blastula stage potential in neural crest cells. Science **348**, 1332–1335 (2015). 25931449 10.1126/science.aaa3655 PMC4652794

[9] A. Lignell, L. Kerosuo, S. J. Streichan, L. Cai, M. Bronner, Identification of a neural crest stem cell niche by Spatial Genomic Analysis. Nat. Commun. **8**, 1830 (2017). 29184067 10.1038/s41467-017-01561-w PMC5705662

[10] V. Mohanty , Folate receptor alpha upregulates Oct4, Sox2 and Klf4 and downregulates miR-138 and miR-let-7 in cranial neural crest cells. Stem. Cells. **34**, 2721–2732 (2016). 27300003 10.1002/stem.2421

[11] P. Scerbo, A. H. Monsoro-Burq, The vertebrate-specific VENTX/NANOG gene empowers neural crest with ectomesenchyme potential. Sci. Adv. **6**, eaaz1469 (2020). 32494672 10.1126/sciadv.aaz1469 PMC7190326

[12] M. L. Basch, M. Bronner-Fraser, M. I. García-Castro, Specification of the neural crest occurs during gastrulation and requires Pax7. Nature **441**, 218–222 (2006). 16688176 10.1038/nature04684

[13] E. Betters, R. M. Charney, M. I. Garcia-Castro, Early specification and development of rabbit neural crest cells. Dev. Biol. **444**, S181–S192 (2018). 29932896 10.1016/j.ydbio.2018.06.012 PMC6685428

[14] M. S. Prasad , Blastula stage specification of avian neural crest. Dev. Biol. **458**, 64–74 (2020). 31610145 10.1016/j.ydbio.2019.10.007 PMC7050198

[15] A. W. Leung , WNT/β-catenin signaling mediates human neural crest induction via a pre-neural border intermediate. Development **143**, 398–410 (2016). 26839343 10.1242/dev.130849 PMC4760313

[16] M. S. Prasad, R. M. Charney, L. J. Patel, M. I. García-Castro, Distinct molecular profile and restricted stem cell potential defines the prospective human cranial neural crest from embryonic stem cell state. Stem. Cell Res. **49**, 102086 (2020). 33370869 10.1016/j.scr.2020.102086 PMC7932500

[17] J. A. Briggs , The dynamics of gene expression in vertebrate embryogenesis at single-cell resolution. Science **360**, eaar5780 (2018). 29700227 10.1126/science.aar5780 PMC6038144

[18] B. A. Schwarz , Prospective isolation of poised iPSC intermediates reveals principles of cellular reprogramming. Cell Stem Cell **23**, 289–305.e5 (2018). 30017590 10.1016/j.stem.2018.06.013 PMC6086589

[19] B. E. Bernstein , A bivalent chromatin structure marks key developmental genes in embryonic stem cells. Cell **125**, 315–326 (2006). 16630819 10.1016/j.cell.2006.02.041

[20] A. Gaspar-Maia, A. Alajem, E. Meshorer, M. Ramalho-Santos, Open chromatin in pluripotency and reprogramming. Nat. Rev. Mol. Cell Biol. **12**, 36–47 (2011). 21179060 10.1038/nrm3036 PMC3891572

[21] D. G. Abernathy , MicroRNAs induce a permissive chromatin environment that enables neuronal subtype-specific reprogramming of adult human fibroblasts. Cell Stem Cell **21**, 332–348 (2017). 28886366 10.1016/j.stem.2017.08.002 PMC5679239

[22] R. L. Judson, J. E. Babiarz, M. Venere, R. Blelloch, Embryonic stem cell–specific microRNAs promote induced pluripotency. Nat. Biotechnol. **27**, 459–461 (2009). 19363475 10.1038/nbt.1535 PMC2743930

[23] D. Subramanyam , Multiple targets of miR-302 and miR-372 promote reprogramming of human fibroblasts to induced pluripotent stem cells. Nature Biotechnol. **29**, 443–448 (2011). 21490602 10.1038/nbt.1862 PMC3685579

[24] F. Anokye-Danso, M. Snitow, E. E. Morrisey, How microRNAs facilitate reprogramming to pluripotency. J. Cell Sci. **125**, 4179–4787 (2012). 23077173 10.1242/jcs.095968 PMC3516433

[25] A. Butler, P. Hoffman, P. Smibert, E. Papalexi, R. Satija, Integrating single-cell transcriptomic data across different conditions, technologies, and species. Nat. Biotechnol. **36**, 411–420 (2018). 29608179 10.1038/nbt.4096 PMC6700744

[26] C. Chazaud , AP-2.2, a novel gene related to AP-2, is expressed in the forebrain, limbs and face during mouse embryogenesis. Mech. Dev. **54**, 83–94 (1996). 8808408 10.1016/0925-4773(95)00463-7

[27] C. P. Chang , Pbx1 functions in distinct regulatory networks to pattern the great arteries and cardiac outflow tract. Development **135**, 3577–3586 (2008). 18849531 10.1242/dev.022350 PMC2680673

[28] B. Murdoch, C. DelConte, M. I. García-Castro, Pax7 lineage contributions to the mammalian neural crest. PloS One **7**, e41089 (2012). 22848431 10.1371/journal.pone.0041089 PMC3407174

[29] T. Sauka-Spengler, M. Bronner-Fraser, A gene regulatory network orchestrates neural crest formation. Nat. Rev. Mol. Cell Biol. **9**, 557–568 (2008). 18523435 10.1038/nrm2428

[30] S. Shibata , Sox10-Venus mice: A new tool for real-time labeling of neural crest lineage cells and oligodendrocytes. Mol. Brain **3**, 1–14 (2010). 21034515 10.1186/1756-6606-3-31 PMC2989948

[31] B. T. Jacques-Fricke, J. Roffers-Agarwal, L. S. Gammill, DNA methyltransferase 3b is dispensable for mouse neural crest development. PLoS One **7**, e47794 (2012), 10.1371/journal.pone.0047794. Epub 2012 Oct 18. 23094090 PMC3475715

[32] I. T. Ling, T. Sauka-Spengler, Early chromatin shaping predetermines multipotent vagal neural crest into neural, neuronal and mesenchymal lineages. Nat. Cell Biol. **21**, 1504–1517 (2019). 31792380 10.1038/s41556-019-0428-9 PMC7188519

[33] R. M., Williams, , Reconstruction of the global neural crest gene regulatory network in vivo. Dev. Cell. **51**, 255–276 (2019). 31639368 10.1016/j.devcel.2019.10.003 PMC6838682

[34] A. Rada-Iglesias , Epigenomic annotation of enhancers predicts transcriptional regulators of human neural crest. Cell Stem Cell. **11**, 633–48 (2012). 22981823 10.1016/j.stem.2012.07.006 PMC3751405

[35] A. S. Hovland , Pluripotency factors are repurposed to shape the epigenomic landscape of neural crest cells. Dev. Cell **57**, 2257–2272 (2022). 36182685 10.1016/j.devcel.2022.09.006 PMC9743141

[36] L. Teng, N. A. Mundell, A. Y. Frist, Q. Wang, P. A. Labosky, Requirement for Foxd3 in the maintenance of neural crest progenitors. Development **135**, 1615–1624 (2008). 18367558 10.1242/dev.012179 PMC2562748

[37] D. Bhattacharya, A. P. Azambuja, M. Simoes-Costa, Metabolic reprogramming promotes neural crest migration via yap/tead signaling. Dev. Cell **53**, 199–211 (2020). 32243782 10.1016/j.devcel.2020.03.005 PMC7236757

[38] E. Van Otterloo, H. Li, K. L. Jones, T. Williams, AP-2α and AP-2β cooperatively orchestrate homeobox gene expression during branchial arch patterning. Development **145**, dev157438 (2018). 29229773 10.1242/dev.157438 PMC5825845

[39] A. Ventura , Targeted deletion reveals essential and overlapping functions of the miR-17∼ 92 family of miRNA clusters. Cell **132**, 875–886 (2008). 18329372 10.1016/j.cell.2008.02.019 PMC2323338

[40] M. Zhou, J. Ma, S. Chen, X. Chen, X. Yu, MicroRNA-17-92 cluster regulates osteoblast proliferation and differentiation. Endocrine **45**, 302–310 (2014). 23673870 10.1007/s12020-013-9986-y

[41] J. K. Eberhart , Microrna 140 modulates pdgf signaling during palatogenesis. Nat. Genet. **40**, 290–298 (2008). 18264099 10.1038/ng.82 PMC2747601

[42] R. J., , Parchem, miR-302 is required for timing of neural differentiation, neural tube closure, and embryonic viability. Cell Rep. **12**, 760–773 (2015). 26212322 10.1016/j.celrep.2015.06.074 PMC4741278

[43] D. Meulemans, M. Bronner-Fraser, Gene-regulatory interactions in neural crest evolution and development. Dev. Cell **7**, 291–299 (2004). 15363405 10.1016/j.devcel.2004.08.007

[44] Y. Mori-Akiyama, H. Akiyama, D. H. Rowitch, B. de Crombrugghe, Sox9 is required for determination of the chondrogenic cell lineage in the cranial neural crest. Proc. Natl. Acad. Sci. U.S.A. **100**, 9360–9365 (2003). 12878728 10.1073/pnas.1631288100 PMC170923

[45] H. Akiyama, M. C. Chaboissier, J. F. Martin, A. Schedl, B. de Crombrugghe, The transcription factor Sox9 has essential roles in successive steps of the chondrocyte differentiation pathway and is required for expression of Sox5 and Sox6. Genes. Dev. **16**, 2813–2828 (2002). 12414734 10.1101/gad.1017802 PMC187468

[46] A. E. Lewis, H. N. Vasudevan, A. K. O’Neill, P. Soriano, J. O. Bush, The widely used Wnt1-Cre transgene causes developmental phenotypes by ectopic activation of Wnt signaling. Dev. Biol. **379**, 229–234 (2013). 23648512 10.1016/j.ydbio.2013.04.026 PMC3804302

[47] D. L. Stemple, D. J., Anderson Isolation of a stem cell for neurons and glia from the mammalian neural crest. Cell **71**, 973–985 (1992), 10.1016/0092-8674(92)90393-q. 1458542

[48] S. G. Gonzalez Malagon , Dissection, culture and analysis of primary cranial neural crest cells from mouse for the study of neural crest cell delamination and migration. J. Vis. Exp. **152**, e60051 (2019), 10.3791/60051. PMC7136076 31633677

[49] M. Simões-Costa, M. E. Bronner, Insights into neural crest development and evolution from genomic analysis. Genome. Res. **23**, 1069–1080 (2013). 23817048 10.1101/gr.157586.113 PMC3698500

[50] P. A. Trainor, B. T. Andrews, Facial dysostoses: Etiology, pathogenesis and management. Am. J. Med. Genet. C Semin. Med. Genet. **163**, 283–294 (2013). 10.1002/ajmg.c.31375 PMC3870197 24123981

[51] E. H. Barriga, P. A. Trainor, M. Bronner, R. Mayor, Animal models for studying neural crest development: Is the mouse different? Development **142**, 1555–1560 (2015). 25922521 10.1242/dev.121590 PMC6514397

[52] D. Nichols, Neural crest formation in the head of the mouse embryo as observed using a new histological technique. J. Embryol. Exp. Morphol. **64**, 105–120 (1981). 7031165

[53] D. Nichols, Formation and distribution of neural crest mesenchyme to the first pharyngeal arch region of the mouse embryo. J. Embryol. Exp. Morphol. **176**, 221–231 (1986). 10.1002/aja.1001760210 3739949

[54] M. Minoux , Gene bivalency at Polycomb domains regulates cranial neural crest positional identity. Science **355**, eaal2913 (2017). 28360266 10.1126/science.aal2913

[55] M. Thomson , Pluripotency factors in embryonic stem cells regulate differentiation into germ layers. Cell **145**, 875–889 (2011). 21663792 10.1016/j.cell.2011.05.017 PMC5603300

[56] E. D. Sperry , The chromatin remodeling protein CHD7, mutated in CHARGE syndrome, is necessary for proper craniofacial and tracheal development. Dev. Dyn. **243**, 1055–1066 (2014). 24975120 10.1002/dvdy.24156 PMC4160830

[57] H. Okuno , CHARGE syndrome modeling using patient-iPSCs reveals defective migration of neural crest cells harboring CHD7 mutations. Elife. **6**, e21114 (2017). 29179815 10.7554/eLife.21114 PMC5705211

[58] R. Bajpai , CHD7 cooperates with PBAF to control multipotent neural crest formation. Nature **463**, 958–962 (2010). 20130577 10.1038/nature08733 PMC2890258

[59] P. Du , An intermediate pluripotent state controlled by microRNAs is required for the naive-to-primed stem cell transition. Cell Stem Cell **22**, 851–864.e5 (2018). 29804889 10.1016/j.stem.2018.04.021 PMC5990487

[60] Y. Wang , Embryonic stem cell–specific microRNAs regulate the G1-S transition and promote rapid proliferation. Nat. Genet. **40**, 1478–1483 (2008). 18978791 10.1038/ng.250 PMC2630798

[61] Y. Wang, R. Medvid, C. Melton, R. Jaenisch, R. Blelloch, DGCR8 is essential for microRNA biogenesis and silencing of embryonic stem cell self-renewal. Nat. Genet. **39**, 380–385 (2007). 17259983 10.1038/ng1969 PMC3008549

[62] C. Melton, R. L. Judson, R. Blelloch, Opposing microRNA families regulate self-renewal in mouse embryonic stem cells. Nature **463**, 621–626 (2010). 20054295 10.1038/nature08725 PMC2894702

[63] H. B. Houbaviy, M. F. Murray, P. A. Sharp, Embryonic stem cell-specific MicroRNAs. Dev. Cell **5**, 351–358 (2003). 12919684 10.1016/s1534-5807(03)00227-2

[64] A. Jouneau , Naive and primed murine pluripotent stem cells have distinct miRNA expression profiles. RNA **18**, 253–264 (2012). 22201644 10.1261/rna.028878.111 PMC3264912

[65] R. J. Parchem , Two miRNA clusters reveal alternative paths in late-stage reprogramming. Cell Stem Cell **14**, 617–631 (2014). 24630794 10.1016/j.stem.2014.01.021 PMC4305531

[66] M. R. Suh , Human embryonic stem cells express a unique set of microRNAs. Dev. Biol. **270**, 488–498 (2004). 15183728 10.1016/j.ydbio.2004.02.019

[67] B. M. Stadler , Characterization of microRNAs involved in embryonic stem cell states. Stem Cells Dev **19**, 935–950 (2010). 20128659 10.1089/scd.2009.0426 PMC3128320

[68] J. Copeland, M. Simoes-Costa, Post-transcriptional tuning of FGF signaling mediates neural crest induction. Proc. Natl. Acad. Sci. U.S.A. **117**, 33305–33316 (2020). 33376218 10.1073/pnas.2009997117 PMC7777031

[69] C. V. Baker, M. Bronner-Fraser, N. M. Le Douarin, M. A. Teillet, Early-and late-migrating cranial neural crest cell populations have equivalent developmental potential in vivo. Development **124**, 3077–3087 (1997). 9272949 10.1242/dev.124.16.3077

[70] N. A. Wall, B. L. Hogan, Expression of bone morphogenetic protein-4 (BMP-4), bone morphogenetic protein-7 (BMP-7), fibroblast growth factor-8 (FGF-8) and sonic hedgehog (SHH) during branchial arch development in the chick. Mech. Dev. **53**, 383–392 (1995). 8645604 10.1016/0925-4773(95)00453-x

[71] E. Veitch, J. Begbie, T. F. Schilling, M. M. Smith, A. Graham, Pharyngeal arch patterning in the absence of neural crest. Curr. Biol. **9**, 1481–1484 (1999). 10607595 10.1016/s0960-9822(00)80118-9

[72] A. Neubüser, H. Peters, R. Balling, G. R. Martin, Antagonistic interactions between FGF and BMP signaling pathways: A mechanism for positioning the sites of tooth formation. Cell **90**, 247–255 (1997). 9244299 10.1016/s0092-8674(00)80333-5

[73] C. Alexander, S. Piloto, P. Le Pabic, T. F. Schilling, Wnt signaling interacts with Bmp and Edn1 to regulate dorsal-ventral patterning and growth of the craniofacial skeleton. PLOS Genet. **10**, e1004479 (2014). 25058015 10.1371/journal.pgen.1004479 PMC4109847

[74] J. Xu , Hedgehog signaling patterns the oral-aboral axis of the mandibular arch. Elife **8**, e40315 (2019). 30638444 10.7554/eLife.40315 PMC6347453

