# Supplementary material for: Post-transcriptional regulation in cranial neural crest cells expands developmental potential
Source: Proc Natl Acad Sci U S A. 2023 Feb 1;120(6):e2212578120. doi: 10.1073/pnas.2212578120 (PMC9963983; doi:10.1073/pnas.2212578120)
Supplement: Supplementary file 1 — Appendix 01 (PDF) [file pnas.2212578120.sapp.pdf]

Supplementary Materials for

Post-transcriptional regulation of cranial neural crest cells expands developmental potential

Rachel A. Keuls, Young Sun Oh, Ivanshi Patel, and Ronald J. Parchem

Correspondence to: Ronald.Parchem@bcm.edu

This PDF file includes:

Materials and Methods
Supplementary Text
Figs. S1 to S7
Tables S1 to S2
Supplementary References

Materials and Methods

Single cell mRNA and ATAC sequencing

Mir-302-eGFP reporter mice, in which the coding sequence for eGFP replaced the miR-302 locus (*41*) were intercrossed to obtain miR-302$^{WT/WT}$ (wildtype), miR-302$^{GFP/WT}$ (heterozygous *mir-302* mutant) and miR-302$^{GFP/GFP}$ (homozygous *mir-302* mutant) embryos. MiR-302$^{WT/WT}$ (wildtype) and miR-302$^{GFP/GFP}$ (homozygous *mir-302* mutant) embryos were harvested at E7.5, E8.25, E8.5, and E9.5. The embryos were decapitated at the otic placode and the cranial region was enzymatically dissociated with papain at room temperature. A single-cell suspension was achieved with gentle pipetting. An equal volume of FBS was then immediately added to quench the enzyme. Following filtration of the single-cell suspension, cells were pelleted at 300g for 5 minutes and resuspended in DMEM with 10% FBS. Cell number and quality was assessed using a hemocytometer and 0.4% Trypan Blue was used as to stain for cell viability (Thermo Fisher). Viability was greater than or equal to 98%. To prepare nuclei for ATAC sequencing, the cell pellet was resuspended in lysis buffer (10mM Tris-Cl pH 7.4, 10mM NaCl, 3mM $MgCl_2$, 0.1% Tween-20, 0.01% Nonidet P40, 0.01% Digitonin, 1% BSA). Cells were lysed for 2 minutes at room temperature and subsequently diluted with wash buffer (10mM Tris-Cl pH 7.4, 10mM NaCl, 3mM $MgCl_2$, 0.1% Tween-20, 1% BSA, 0.1% Tween-20). Nuclei were harvested at 500g for 5 minutes, washed, pelleted, and subsequently resuspended in nuclei buffer (10x Genomics 2000153). Nuclei were stained with Trypan Blue and were quantified using a hemocytometer. Generation of gel beads-in-emulsion (GEM) was performed on a 10x Chromium Controller using the Chromium Next GEM Chip G for mRNA (10x Genomics

1000120) and Chip H for ATAC (10x Genomics 1000161). Libraries were prepped for mRNA using the Chromium Next GEM Single Cell 3' Library Kit v3.1 (10x Genomics 1000158) and for ATAC using the Chromium Next GEM Single Cell ATAC Library Kit v1 (10x Genomics 1000163). The Chromium Single Index Kit T Set A (10x Genomics 1000213) was used for mRNA library indexing and i7 Multiplex Kit N Set A (10x Genomics 1000084) was used for ATAC library indexing. The resulting libraries were validated using the Agilent High Sensitivity NGS Fragment Analysis Kit on a 12-Capillary Fragment Analyzer. Libraries were subjected to paired-end sequencing on an Illumina NextSeq platform following 10x Genomics recommendations.

Single-cell mRNA and ATAC data analysis

For single-cell mRNA analysis, raw .BCL files were downloaded using Illumina's BaseSpaceCLI version 0.10.7. The 10x Genomics Cell Ranger mkfastq version (v3.0.2) converted .BCL to .FASTQ files. The resulting .FASTQ files were aligned to the mm10 genome using the 10x Genomics Cell Ranger count (v3.0.2) with mapping rates between 86-90%. We detected approximately 17,000-19,000 total genes in each sample. Data were processed and visualized using Seurat version 3.1.5. Mitochondrial genes were filtered out. Clustering was performed in Seurat version 3.1.5 (*25*), using statistically significant principal components chosen based on their standard deviation. Cells of low complexity with less than 200 UMIs and doublets/triplets with greater than 2500 UMIs were filtered out. Data were log-normalized and integrated for a *miR-302*$^{-/-}$ versus *miR-302*$^{+/+}$ comparison. Visualization of the cells was performed using the Uniform Manifold Approximation and Projection for Dimension Reduction (UMAP) algorithm as implemented by Seurat. Cell clusters were identified using the correct number of statistically significant principal components based on expression of known markers as previously described (*75*). Differential gene expression analyses were performed using Seurat's FindMarkers function, and gene ontology was performed using Enrichr's Biological GO. Pseudotime analysis was performed using Monocle v2.18.0 (*76-78*).

For single-cell ATAC data, raw .BCL files were downloaded using Illumina's BaseSpaceCLI version 0.10.7 and 10x Genomics Cell Ranger ATAC mkfastq (version 1.1.0 or version 2.0) was used to convert BCL to FASTQ files. Peaks were unified, quantified, and visualized in Seurat (v4.1.1) and Signac (version 1.7.0), and single-cell ATAC data was overlayed with cell populations from annotated single-cell mRNA similar to previous studies (*25*). The UnifyPeaks command with the reduce function from the GenomicRanges package was used to extract peak coordinates from each Seurat object. The GetFragments command

was used to quantify peaks and the resulting matrix was added to the object using CreateAssayObject. Peak datasets were merged for corresponding *miR-302*$^{-/-}$ and *miR-302*$^{+/+}$embryos at each age. Term frequency inverse document frequency (TF-IDF) normalization and partial singular value decomposition using LSI reduction was performed for each merged dataset. The correlation between each LSI component and sequencing depth was assessed with DepthCor and there was a strong correlation between the first LSI component and the total number of counts. Therefore, clustering was performed using components 2:30 for the RunUMAP, FindNeighbors, and FindClusters algorithms as implemented by Seurat. Datasets were merged for corresponding *miR-302*$^{-/-}$ and *miR-302*$^{+/+}$ embryos at each age, as well as across developmental time. TSS enrichment score for each cell was determined using the TSSEnrichment function in Signac. Granges objects were constructed from EnsDb.Mmusculus.v79. Gene activity was quantified by summing the reads intersecting the gene body and promoter region (2000bp upstream of TSS). A gene activity matrix was added to each object and log-normalized. Subsequently, preprocessed single-cell mRNA data was added to each object using the FindTransferAnchors with CCA reduction, and data was transferred using TransferData with LSI weight reduction including components 2:30. The RegionStats function was used to assess G/C content, sequence length, and dinucleotide frequency. The FindMarkers function was used to identify differentially accessible peaks; motif enrichment was identified using FindMotifs. For gene lists, motifs were called within 2kb of the loci of interest by Homer. Enrichr's Biological Process was used for gene ontology analyses. Gene lists were obtained from the Broad Institute Molecular Signatures Database (https://www.gsea-msigdb.org/gsea/msigdb/genesets.jsp?collection=C5). MiRNA targets were identified using TargetScanMouse 7.2 (*79*). Cartoons and schematics were produced using BioRender illustrations.

<u>Embryo processing and immunofluorescence</u>

Mouse embryos were dissected in phosphate buffered saline (PBS) at pH 7.4 and fixed overnight at 4ºC in 3.7% formaldehyde diluted in PBS. Following fixation, embryos were washed with PBS containing 0.1% Triton (PT), stored in methanol at -30ºC, and rehydrated in PT before use. For cryosectioning, embryos were cryopreserved by equilibrating in 10%, 20%, 30% w/v sucrose in PT, followed by 1:1 30% sucrose:OCT Compound (Fisher Scientific 23730571) and lastly in 100% OCT. Embryos were embedded in OCT, flash frozen in a dry ice and 100% ethanol bath, and stored at -80ºC until sectioning. 10µm thick sections were adhered to glass slides, and slides were stored at -80ºC until staining. Embryos for whole mount and sections on

slides for immunofluorescence were washed with PT and blocked for 1 hour at room temperature (RT) in 5% Gibco normal goat serum (16210064) and 1% bovine serum albumin (Fisher Scientific BP1600100). Primary antibodies were diluted in blocking buffer and applied to tissue overnight at 4ºC (PAX3 1:1000 [DSHB PAX3]; PAX7 1:100 [DSHB PAX7]; TFAP2A 1:100 [DSHB 3B5]; SOX9 [EMD Millipore AB5535]; TUJ1 1:1000 [BioLegend 801202]). Secondary antibodies (AlexaFluor) in blocking buffer were applied for 1.5 hours at room temperature. GFP signal was amplified for reporter mouse cross sections using GFP booster (Bulldog Bio BGA488). Sections were mounted with Fluoromount G (Fisher Scientific OB10001). Images of cross sections were taken on Leica DMI6000B, Zeiss LSM980, or Zeiss LSM780. Whole mount embryos were imaged on a Leica M165FC dissecting microscope with a Leica DFC 3000G camera, Zeiss LSM780, or Zeiss LSM980.

In situ hybridization

Wholemount embryos were re-fixed for 20 min at room temperature using formaldehyde diluted in PBS to a final concentration of 4%. Following re-fixation, tissue was washed in PBS and incubated in hybridization buffer (5× SSC [pH 4.5], 1% SDS, 50% Formamide) at 37ºC for 2 hours. Probes were diluted 1:500 in hybridization buffer and incubated for 2 hours at 37ºC. After probe removal, embryos were washed several times in hybridization buffer at 37ºC for 2 hours. Embryos were then washed several times in PT at room temperature for 1 hour. This was followed by incubating overnight with antibody at 4ºC (Boehringer-Mannheim anti-digoxigenin/fluorescein-alkaline phosphatase [AP]) diluted (1:3000) in PT overnight at 4ºC. After incubation, the embryos were washed in PT for 1 hour before reacting with BM-purple for at 2 hours at room temperature.

Embryo dissociation and cell sorting

Wnt1-Cre (*45*) or Sox10-Cre mice (*80*) were crossed with LSL-Td-Tomato (*81*) to lineage trace murine neural crest cells at E8.5 and E9.5 respectively. Mouse embryos were decapitated above the otic placode; extraembryonic structures were removed from E7.5 mouse embryos. The cranial region or E7.5 epiblast was enzymatically dissociated with papain at room temperature combined with gentle pipetting until a single cell suspension was achieved. At this time, an equal volume of FBS was added immediately to quench the enzyme. After filtration of the single cell suspension, cells were pelleted at 300g for 5 minutes and resuspended in FACS Buffer (PBS with 1% bovine serum albumin). Samples were filtered, stained with DAPI, and the

fluorescent cells were sorted on a BD FACSAria III instrument with a 70-µm nozzle into a 1.5ml Eppendorf tube containing 500µl Trizol-LS (Thermo Fisher 10296028).

Bulk RNA isolation, library preparation, and sequencing

Total RNA was extracted from cells using the RNeasy Micro Kit (QIAGEN 74004). Small RNA libraries were made from 500pg RNA using the NEXTflex Small RNA-Seq kit v3 (Bioo Scientific NOVA5132). Libraries were gel purified via TBE-PAGE and quantified using the High Sensitivity NGS Fragment Analysis Kit (Agilent, formerly AATI DNF-474-0500) on a 12-Capillary Fragment Analyzer. For mRNA sequencing, the SMART-Seq Ultra Low Input RNA Kit for Sequencing (Takara 634889) was used to generate cDNA from approximately 500pg total RNA. The High Sensitivity NGS Fragment Analysis Kit was used to validate cDNA size and the Quant-iT dsDNA Assay Kit, high sensitivity (Thermo Fisher Q33120) was used to quantify concentration. 100pg of cDNA was tagmented and ligated using the Nextera XT DNA Library Kit (Illumina FC-131-1024) at ½ volumes to produce sequencing libraries. The resulting libraries were validated and quantified as described above for cDNA. Equal concentrations (2 nM) of libraries were pooled and subjected to paired-end (2x75) sequencing of approximately 15 million reads per sample using the Mid Output v2 kit (Illumina FC-404-2001) on a NextSeq550 following the manufacturer's instructions.

Bulk small-RNA and mRNA sequencing bioinformatic analysis

Sequencing files from each flow cell lane were downloaded and the resulting .FASTQ files were merged. FastQC (v0.10.1) was used for quality control. Reads were mapped to the mouse genome mm10 assembly using STAR (v2.5.0a). In R (v3.5.2), gene count matrices were built with Bioconductor packages Rsamtools (v2.0.0) and GenomicFeatures (v1.32.2). mRNA-sequencing datasets were annotated with UCSC transcripts downloaded from Illumina iGenomes in GTF file format, and small RNA sequencing datasets were annotated with the miRBase *Mus musculus.*gff3. We determined reads per million (RPM) using GenomicAlignments (v1.16.0). Principal component analysis (PCA) was performed using an rlog transformed gene expression matrix of global gene expression >1 for each region. DESeq2 (v1.20.0) was used for differential gene expression analysis and read count normalization. miRNA predicted targets were obtained from TargetScan, and any gene with a predicted binding site for a given miRNA within the 3'UTR was called a predicted target. Expression heat maps were generated using ComplexHeatmap (v2.0.0). Clustering was done using the Mfuzz package, and the replicates were averaged to create the expression matrix, which was filtered

to remove 'NA'. Fuzzy c-means clustering was used with the estimated m fuzzifier value for each dataset. Clusters obtained from the small RNA-seq dataset were re-ordered to match the clusters obtained from the RNA-seq dataset. Biological Process GO analyses were determined using Enrichr and visualized using ggplot and GOplot.

Luciferase assay

For Sox9, three miR302a-5p binding sites 'TTTAAG' were tested for target verification alongside Fgf15 as a positive control using miR302b-3p binding site 'GCACTTA'. Sox9 binding sites were mutated from 'TTTAAG' to 'TTGACG' and for Fgf15 from 'GCACTTA' to 'GAAATTA'. 200-300bps of the 3'UTRs of respective targets spanning the respective binding sites were amplified from mouse genomic DNA cells using Phusion polymerase and individually sub-cloned into psiCHECK-2 vector (Promega) and mutated using the T5 exonuclease DNA assembly protocol (*82*) downstream of Renilla luciferase. 293T cells were plated in a 0.1% gelatin pre-coated 96 well plate 2 days prior to transfection at 5000 cells/well density. Cells were transfected at 60-80% confluency with 20ng of plasmid DNA with or without 500nM of respective miRIDIAN miRNA mimics (Dharmacon) using Lipofectamine 2000 transfection reagent following manufacturer's protocol. Cells were processed 48 hours post transfection using the Dual Glo luciferase assay system (Promega E2920) following the manufacturer's protocol to measure luminescence using the PerkinElmer Victor X3 plate reader. The normalized activity of each target was computed using robust errors-in-variable regression (REIV) (*83*) with a significance threshold of 0.05 and standard mean of error. One-way ANOVA was used to determine the significance between control, 3'UTR+mimic, and mutant 3'UTR+mimic.

Generation of *mir-302*$^{-/-}$ mutant mouse

CRISPR/Cas9 genome editing was used to delete the miR-302 cluster. Cas9 mRNA was purchased from IDT, Inc. and together with sgRNAs that flank the miR-302 cluster (gRNA 1 forward primer: 5'- caccGGCTCCCTTCAACTTTAACA-3'; gRNA 1 reverse primer: 5' – aaacTGTTAAAGTTGAAGGGAGCC – 3'; gRNA 2 forward primer: 5' – caccgGTTAACGCAATTGCTGATT – 3'; gRNA 2 reverse primer: 5' – aaacAATCAGCAATTGCGTTAACc – 3') were microinjected into single-cell C57BL/6J mouse zygotes by the Baylor College of Medicine Mouse Embryonic Stem Cell Core. Mice with putative correct targeting were identified using miR-302 genotyping primers listed in table s2 and were then bred to C57BL/6J mice and F1 offspring were sequenced to confirm genotype. Prior to

phenotyping studies, F1 animals were backcrossed to C57BL/6J for 10 generations. *Mir-302*$^{+/-}$ mice were interbred to obtain *mir-302*$^{-/-}$ embryos and small RNA sequencing was used to further confirm the loss of *mir-302* while mRNA sequencing was used to demonstrate no change in expression of *Larp7*, the *mir-302* host gene.

<u>*Sox9* deletion and generation of *mir-302*; *Sox9* double knockout embryos</u>

To generate *Sox9*$^{-/-}$ embryos, *Sox9*$^{flox/flox}$ mice (*44*), were bred with Wnt1-Cre mice (*45*) to generate Wnt1-Cre+; Sox9$^{flox/+}$ female mice which were then crossed with *Sox9*$^{flox/flox}$ or *Sox9*$^{flox/+}$ male mice and the resulting litters of embryos were harvested at E8.5 and E9.5. *Sox9*$^{+/+}$ and *Wnt1-Cre+; Sox9*$^{flox/flox}$ embryos were assessed for neural crest development using immunofluorescence of wholemount embryos and transverse cross sections through the cranial region for PAX3, PAX7, and SOX9, as described above. To generate *mir-302*$^{-/-}$; *Sox9*$^{-/-}$ embryos, *mir-302*$^{+/-}$ animals were crossed with *Sox9*$^{flox/flox}$ mice and *Sox9*$^{flox/+}$; *Wnt1-Cre*+ mice to obtain *mir-302*$^{+/-}$; *Sox9*$^{flox/flox}$ males and *miR-302*$^{+/-}$; *Sox9*$^{flox/flox}$; *Wnt1-Cre*+ female animals which were then interbred, and the resulting embryos were harvested at E9.5. Transverse cross sections through the cranial region were assessed for neural crest development as described above using immunofluorescence for PAX3 and TFAP2A. For single-cell mRNA sequencing, the embryonic cranial region was harvested and processed as described above.

<u>Mouse strains and genotyping</u>

All research and animal care procedures were approved by the Baylor College of Medicine Institutional Animal Care and Use Committee. Animals were housed in the Association for Assessment and Accreditation of Laboratory Animal Care-approved animal facility at Baylor College of Medicine. All strains were maintained on C57BL6 background. For adult mouse genotyping, 1-2mm ear clips were obtained and lysed using 75ul of a 25mM NaOH and 0.2mM EDTA buffer at 98ºC for 1 hour before neutralization with 75ul of 40mM Tris-Cl, pH 5.5. For embryo genotyping, DNA was isolated from yolk sacs and digested overnight in lysis buffer (50 mM Tris-HCl [pH 8.0], 10 mM EDTA, 100 mM NaCl, 0.1% SDS, and 5mg/ml proteinase K). Cell debris was removed, and an equal amount of isopropanol was used to precipitate DNA at -30ºC for 1 hour. DNA was pelleted by a 30-minute centrifugation, dried, and resuspended in water. PCR for all alleles was performed via touchdown PCR with a total of 40 cycles with annealing at 64ºC, 62ºC, and 60ºC for 5 cycles each, followed by 58ºC for 25 cycles. All PCR primers and expected band sizes are in Table 2.

Fig. S1. Chromatin accessibility increases during cranial neural crest specification.

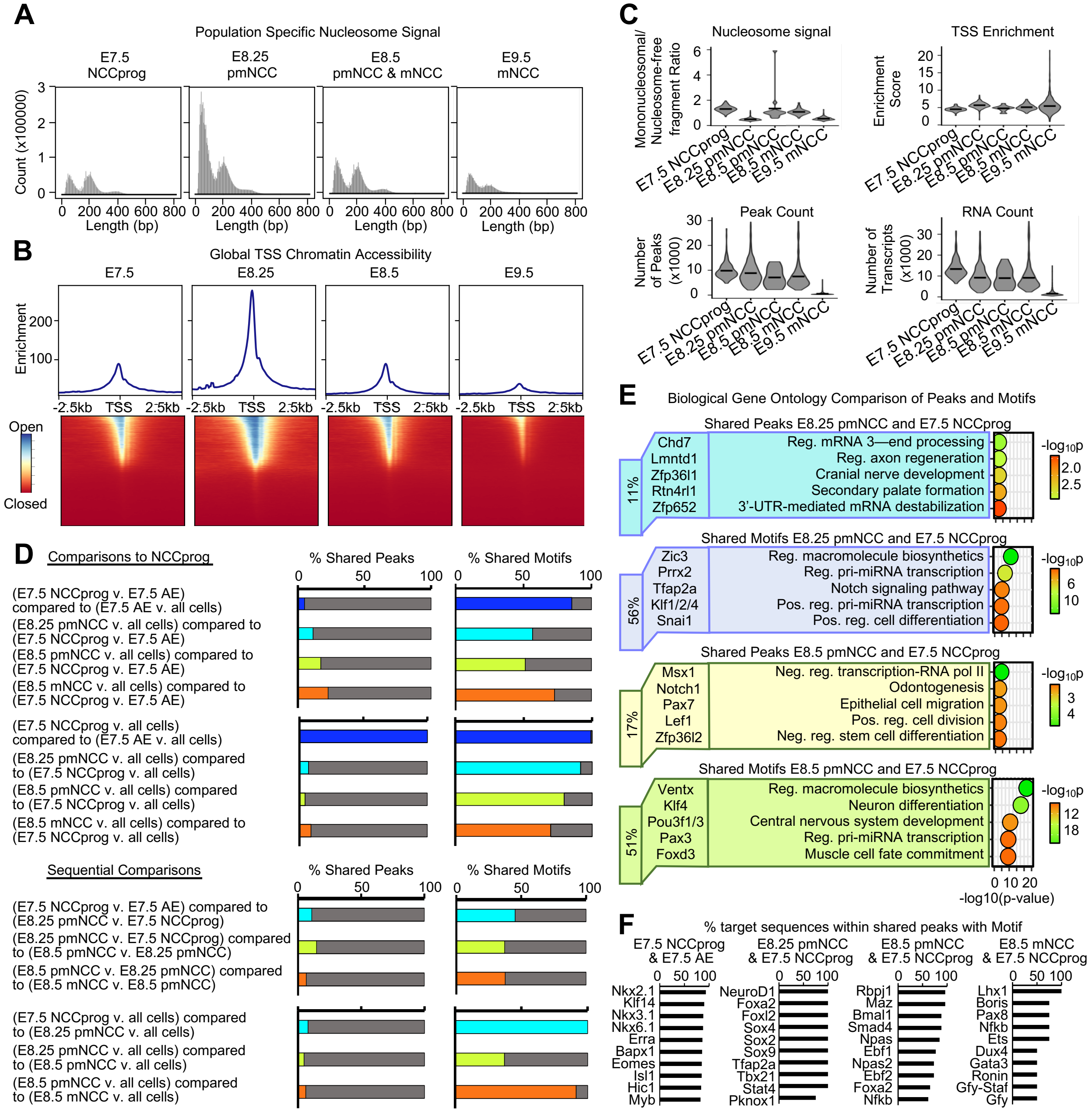

Fig. S1. Chromatin accessibility increases during cranial neural crest specification. (**A**) Nucleosome plot showing count and length of reads for each neural crest-associated cell population across time. Chromatin accessibility increased in the E8.25 premigratory cranial neural crest compared to the neural crest progenitors within the E7.5 anterior ectoderm expressing Sox9, Pax7, Pax3, or Msx1. (**B**) Peak plot and heat map showing global chromatin accessibility at E7.5, E8.5, and E9.5. Chromatin structure around transcriptional start sites is permissive at E7.5 and E8.5 and accessibility declines at E9.5. (**C**) Violin plots showing how nucleosome signal, TSS enrichment, peak counts and RNA counts in each neural crest associated population across time. (**D**) Quantification of comparisons between differentially accessible peaks and enriched motifs (Figure 1 – Source Data 5). All comparisons between peaks and motifs of E8.25 and E8.5 neural crest populations compared to E7.5 neural crest

progenitors are included within Figure 1 – Source Data 6. All sequential comparisons between neural crest associated populations across time are provided in Figure 1 – Source Data 7. (**E**) Biological gene ontology was used to predict function of shared peaks and motifs. Gene ontology analysis is shown for peaks and motifs shared between the E8.25 premigratory neural crest vs. all E8.25 cells and the E7.5 neural crest progenitors vs. the E7.5 anterior ectoderm. Gene ontology analysis is also shown for peaks and motifs shared between the E8.5 premigratory neural crest vs. all cells and the E7.5 neural crest progenitors vs. the E7.5 anterior ectoderm. (**F**) Top 10 motifs enriched in peaks shared between the E7.5 neural crest progenitors and the neural crest populations E8.25 and E8.5. All motifs found within shared peaks provided in Figure 1 – Source Data 8.

Fig. S2. Emergence of ectomesenchymal and neuronal-biased neural crest.

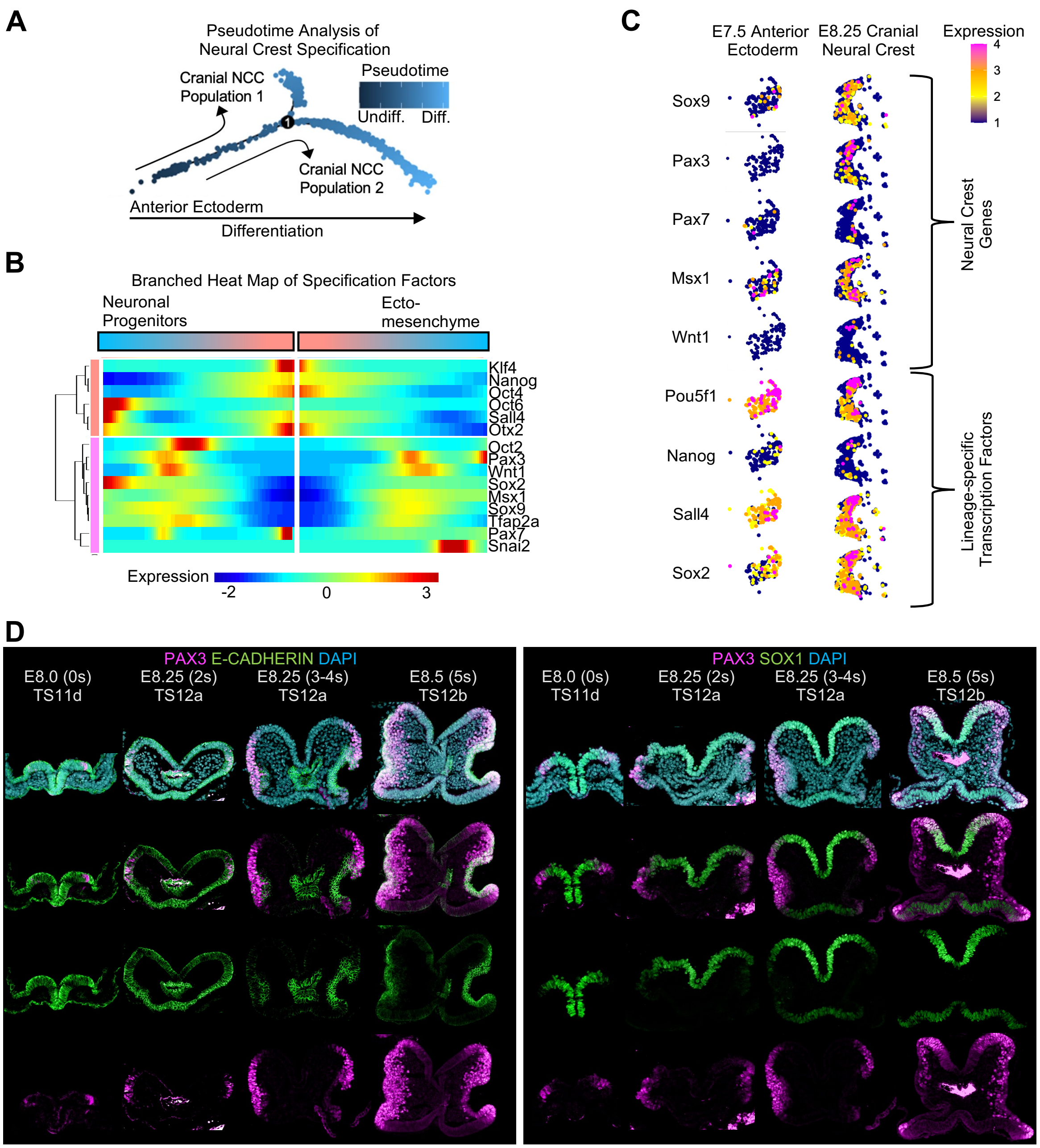

Fig. S2. Emergence of ectomesenchymal and neuronal-biased neural crest.
(**A**) Pseudotime trajectory of E7.5 anterior ectoderm and E8.25 cranial neural crest. Cells are colored according to their progression in pseudotime. (**B**) Branched heat map showing expression of specification genes during formation of each population of cranial neural crest. Most canonical neural crest genes are similarly expressed in each premigratory neural crest population. (**C**) UMAP plots showing expression of lineage-specific transcription factors from early development and canonical neural crest genes as cells transition from E7.5 anterior ectoderm to E8.25 cranial neural crest. (**D**) Full sections of immunofluorescence for PAX3 and E-CADHERIN, as well as PAX3 and SOX1 from E8.0 to E8.5.

Fig. S3. Epiblast and neural crest transcription factors form a regulatory network.

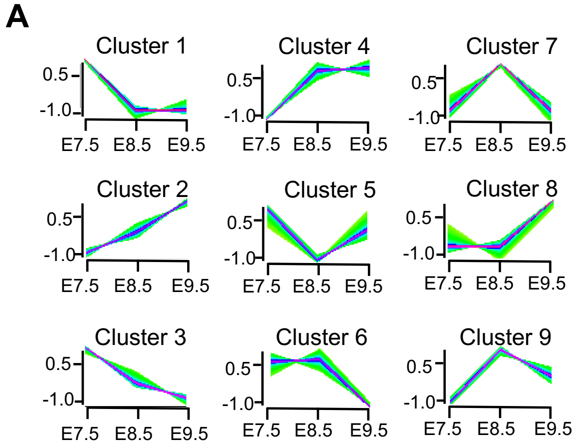

Fig. S3. Epiblast and neural crest transcription factors form a regulatory network.
(**A**) Line plots showing expression of each gene module obtained from unsupervised clustering at E7.5, E8.5, and E9.5. Mfuzz clustering was used to identify gene modules. Genes within the cluster 1 gene module were expressed at E7.5, declined at E8.5 and E9.5, and were referred to as the epiblast gene module. Genes within the cluster 2 gene module increased in expression from E7.5 to E9.5 and were referred to as the neural crest differentiation gene module. Genes within the cluster 4 gene module increased in expression from E7.5 to E8.5 and were maintained from E8.5 to E9.5. Therefore cluster 4 was referred to as the neural crest specification gene module. Genes within the cluster 6 module were expressed at E7.5 and E8.5, declined at E9.5, and were referred to as the multipotency gene module.

Fig. S4. miR-302 is expressed in cranial neural crest cells.

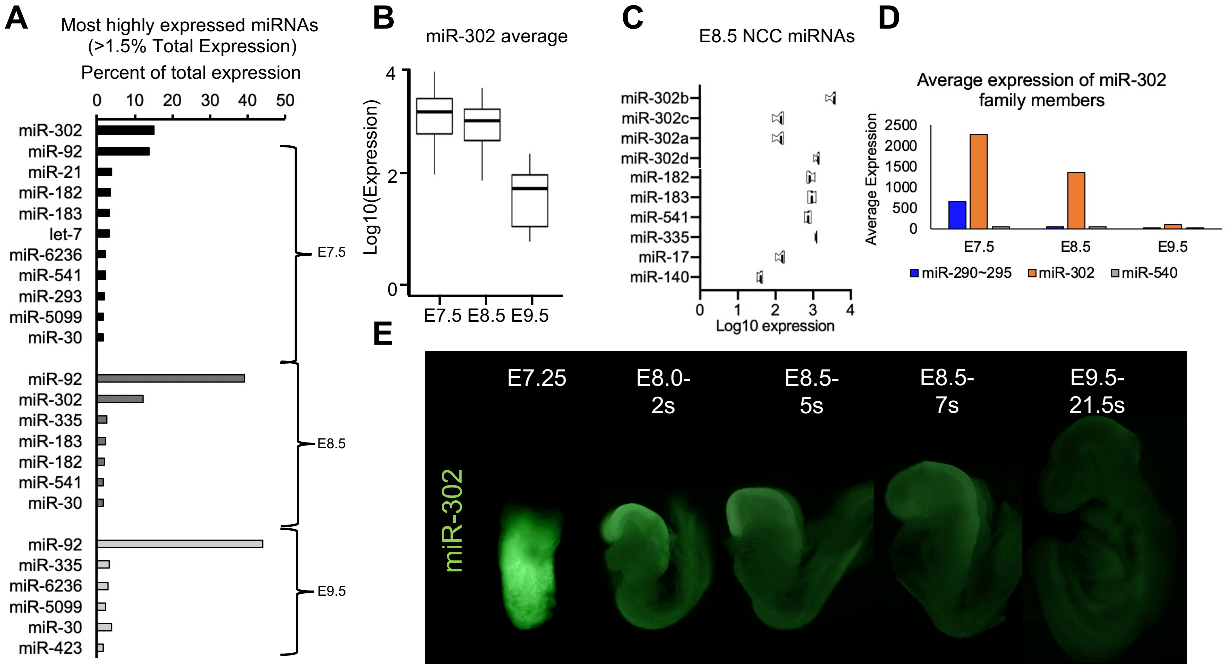

Fig. S4. miR-302 is expressed in cranial neural crest cells.
(**A**) Bar plot showing the most highly expressed microRNAs at each time point. (**B**) Boxplot of miR-302 cluster average expression over time, showing that expression of *miR-302* does not significantly change from E7.5 to E8.5 (ANOVA, p=0.46) and there is a decrease from E8.5 to E9.5 (ANOVA, p=0.00018). (**C**) Violin plot of miRNA expression for prevalent pluripotency and multipotency miRNAs compared to canonical neural crest miRNAs. MiR-302b/c/a/d are more highly expressed than canonical neural crest microRNAs miR-17 and miR-140. (**D**) Bar plot showing the average expression of miR-290/302 family members that share a common seed sequence and have similar predicted targets. MiR-290~295 decreases in expression after E7.5 while miR-302 is maintained. (**E**) MiR-302-eGFP reporter embryos imaged at 200ms exposure from E7.5 to E9.5. MiR-302 expression is maintained more so in the cranial region compared to the trunk until its decline at E9.5.

Fig. S5. *mir-302* differentially regulates specification of ectomesenchymal versus neuronal progenitor neural crest.

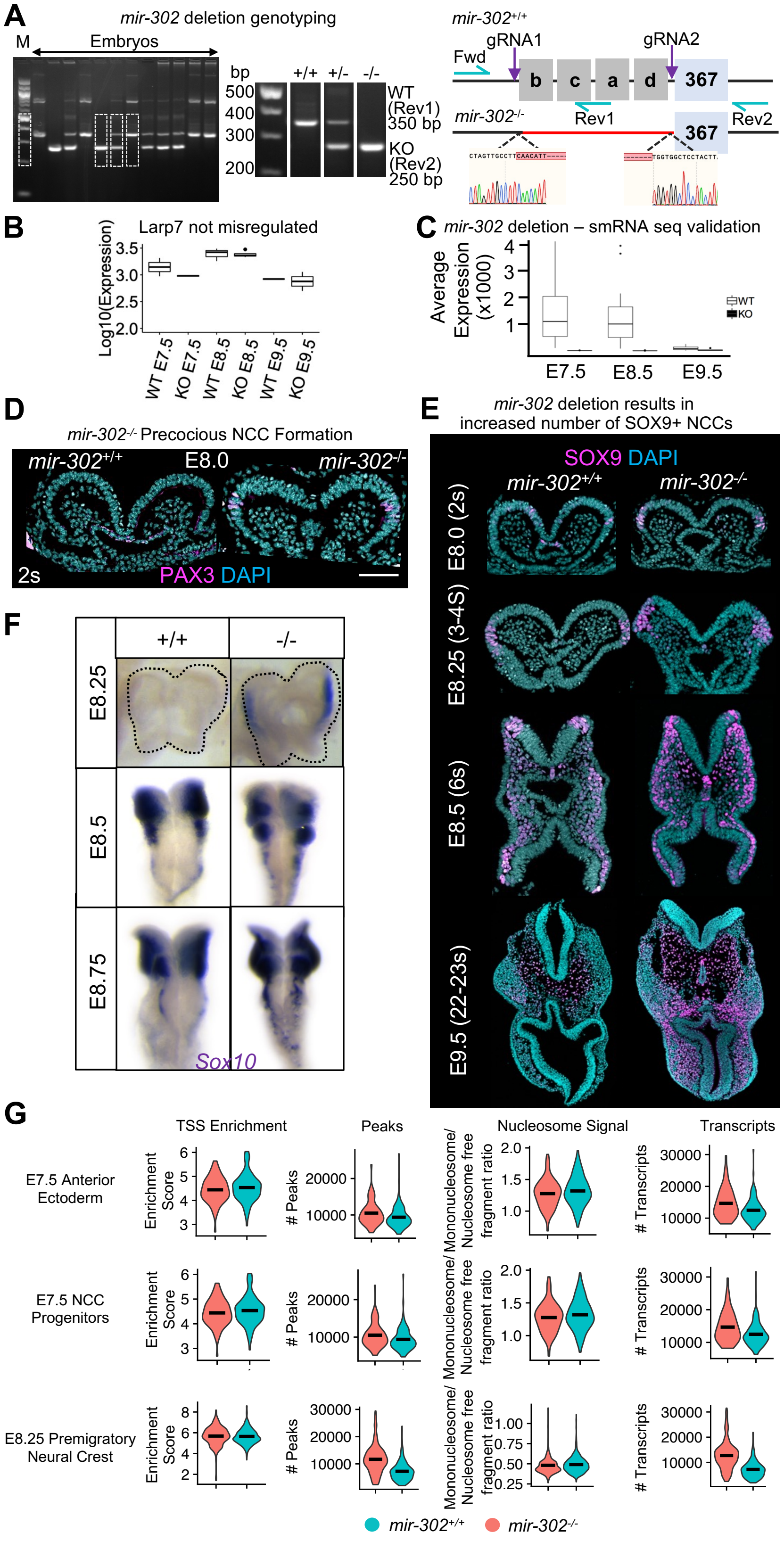

Fig. S5. *mir-302* differentially regulates specification of ectomesenchymal versus neuronal progenitor neural crest.

(**A**) Genotyping for deletion allele and locus map of *mir-302*$^{-/-}$ mouse line. Dashed lines represent insets shown in center panel. (**B**) Boxplot of *Larp7* expression revealing minimal effect on *mir-302* host gene expression in the *mir-302*$^{-/-}$ mouse line. (**C**) Boxplot of miR-302 expression in *mir-302*$^{+/+}$ and *mir-302*$^{-/-}$ embryos over time confirming the deletion of *mir-302*. (**D**) Immunofluorescence for Pax3 at E8.0 in *mir-302*$^{+/+}$ and *mir-302*$^{-/-}$ embryos similarly revealed precocious neural crest formation within the *mir-302*$^{-/-}$. (**E**) Immunofluorescence for SOX9 over time revealed precocious neural crest formation and an increase in SOX9-positive cells over time (**F**) In situ hybridization for migratory neural crest marker *Sox10*. (**G**) Violin plots showing TSS Enrichment, number of peaks, nucleosome signal, and number of transcripts within peaks that passed quality control of the *mir-302*$^{+/+}$ and *mir-302*$^{-/-}$ E7.5 anterior ectoderm, E7.5 NCC progenitors, and E8.25 premigratory neural crest.

Fig. S6. miR-302 targets *Sox9* to restrain specification of the ectomesenchyme lineage.

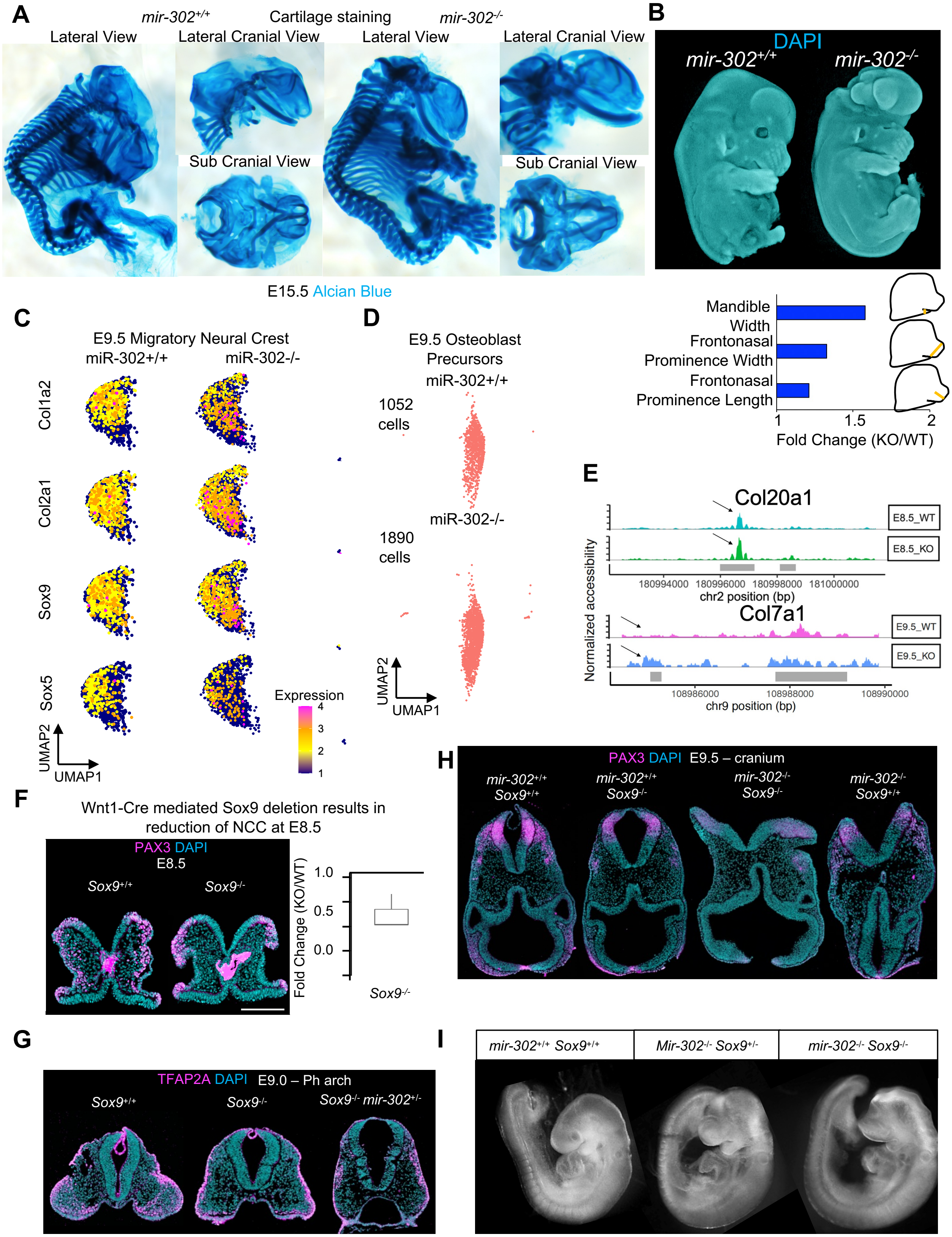

Fig. S6. miR-302 targets *Sox9* to restrain differentiation of the ectomesenchyme lineage of neural crest.

(**A**) Alcian blue staining of E15.5 *mir-302*$^{+/+}$ and *mir-302*$^{-/-}$ embryos showing a minimal change in cartilage formation. (**B**) Comparing craniofacial measurements of wholemount images of *mir-302*$^{+/+}$ and *mir-302*$^{-/-}$ embryos at E15.5. (**C**) Feature plots showing expression of *Col1a2*, *Col2a1*, *Sox9*, and *Sox5* in *mir-302*$^{+/+}$ and *mir-302*$^{-/-}$ E9.5 migratory neural crest. (**D**) Quantification of number of cells in the osteoblast precursor population obtained from E9.5 single-cell mRNA sequencing. (**E**) Coverage plots chondrocyte genes in *mir-302*$^{+/+}$ and *mir-302*$^{-/-}$ embryos at E8.5 and E9.5. (**F**) Immunofluorescence of transverse cross sections of E8.5 (TS12b, 5 somites) *Sox9*$^{+/+}$and *Sox9*$^{-/-}$ embryos and quantification (ANOVA p=0.023). Scale bar is equal to 50 μm. Raw data available in Figure 6 – Source Data 1. (**G**) Immunofluorescence for TFAP2A in cross sections through the pharyngeal arch of *Sox9*$^{-/-}$ and *Sox9*$^{-/-}$; *mir-302*$^{+/-}$ embryos. (**H**) Immunofluorescence for PAX3 in cross sections through the cranial region of *mir-302*$^{+/+}$*; Sox9*$^{+/+}$, *Sox9*$^{-/-}$, *Sox9*$^{-/-}$; *mir-302*$^{-/-}$, and *mir-302*$^{-/-}$ embryos at E9.5. (**I**) Bright field images of *mir-302*$^{+/+}$*; Sox9*$^{+/+}$, *mir-302*$^{-/-}$, and *mir-302*$^{-/-}$; *Sox9*$^{-/-}$ at E9.5.

Fig. S7. miR-302 is required for the differentiation of the peripheral neuron lineage of cranial neural crest.

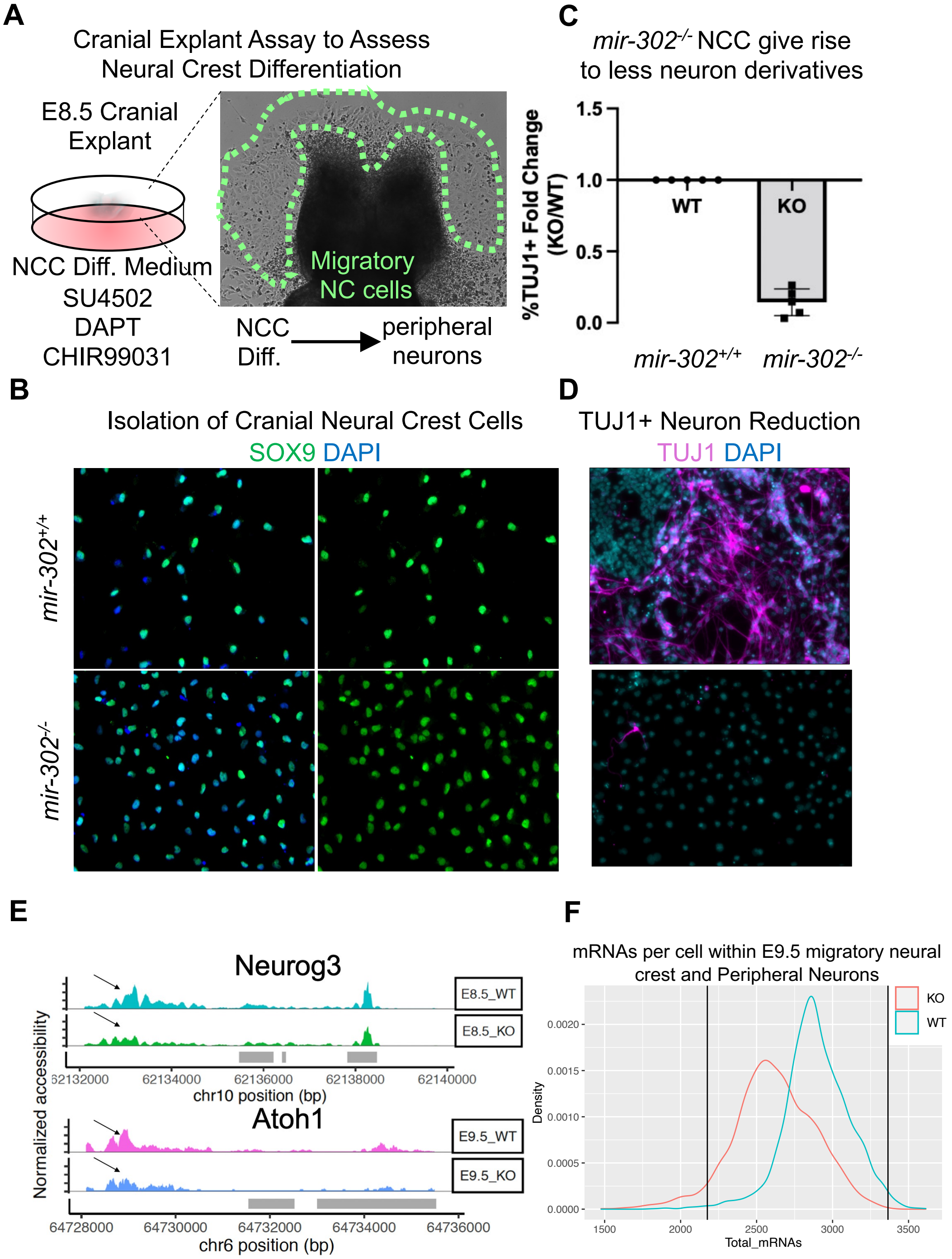

Fig. S7. miR-302 is required for the differentiation of the peripheral neuron lineage of cranial neural crest.
(**A**) Schematic demonstrating that cranial neural crest were isolated from E8.5 cranial explants (**B**) SOX9 staining demonstrating isolation of cranial neural crest cells and removal of non-neural crest tissues. (**C**) Reduction in peripheral neuron differentiation of cranial neural crest cells upon *mir*-302 deletion (N=5 for each genotype). Data were normalized for differences in

total cell number and the percent of cells staining positive for TUJ1 are plotted for *mir-302*$^{+/+}$ and *mir-302*$^{-/-}$ embryos. Raw data provided in Figure 7 – Source Data 1. (**D**) TUJ1 staining of neural crest cells harvested from *mir-302*$^{+/+}$ and *mir-302*$^{-/-}$ embryos. (**E**) Coverage plots showing a reduction in accessibility around neuronal genes upon *mir-302* deletion at E8.5 and E9.5. (**F**) Density plot showing that a reduction in chromatin accessibility is correlated with a reduction in gene expression in *mir-302*$^{-/-}$ peripheral neurons at E9.5.

Table S1.

E7.5									
Anterior Cranial Mesoderm	*T*	*Aplnr*	*Fst*	*Acvr1b*	*Pdgfra*	*Lefty2*	*Tbx6*	*Nkx3.1*	*Bmp4*
Anterior Proximal Embryonic Ectoderm	*Otx2*	*Bcat1*	*Gas1*	*Pou5f1*	*Fgf5*	*Sox2*	*Lefty2*	*Six3*	*Tbx6*
Distal Embryonic Ectoderm	*Otx2*	*Bcat1*	*Gas1*	*Pou5f1*	*Fgf5*	*Krt18*			
Allantois	*Pou5f1*	*Cdh1*	*Fn1*	*Ifitm1*					
Visceral Endoderm	*Cited1*	*Afp*	*Hnf4a*	*Cubn*	*Clic6*	*Rbp4*	*Myo6*	*Ttr*	*Apob/c2/a4/a1*
Paraxial Mesoderm	*Npm1*	*Gsta4*	*Mif*	*Ckb*	*Mesp1*				
Definitive Endoderm	*Sox17*	*Gsc*	*Cxcr4*	*Foxa2*					
Primordial Germ Cells	*Rhox5*	*Fst*							
E8.25, E8.5, E9.5									
Forebrain	*Pax6*	*Foxg1*	*Otx2*	*Sox1*	*Fgf8*				
Midbrain	*Otx2*	*Sox1*	*En1*	*Pax2*					
Hindbrain	*En1*	*Gbx2*	*Hoxa2*	*Hoxa4*	*Sox1*				
Non-Neural Ectoderm	*Cdh1*	*Krt18*	*Krt8*	*Epcam*	*Trp63*	*Grhl2*			
Migratory Neural Crest	*Sox10*	*Sox9*	*Pax3*	*Pax7*	*Tfap2a*				
Premigratory Neural Crest	*Sox9*	*Pax3*	*Pax7*	*Tfap2a*					
Peripheral Neurons	*Pcsk1n*	*Sox9*	*Tubb3*						
Prechordal Region	*T*	*Shh*	*Isl1*	*Chrd*	*Foxa2*				
Megakaryocytes	*Plekhf2*	*Itgb3*	*Itga2b*	*Plek*	*Pf4*				
Smooth Muscle	*Cd63*	*Myl6*	*Talgn*	*Lgals1*	*Fth1*	*Tpm1/2*	*Myl9*	*Actg1*	
Osteoblast Precursors	*Rbm8a*	*Col11a1*	*Col2a1*	*Foxp2*	*Sox9*	*Epha7*			
Mesoderm	*T*	*Aplnr*	*Fst*	*Acvr1b*	*Pdgfra*	*Meox1*	*Mixl1*		
Neurons	*Tubb3*	*Nrn1*	*Fgf15*						
Notochord	*Shh*	*Slit1*	*Ntn1*	*Noto*					
Endoderm	*Sox17*	*Pecam1*	*Ptprb*	*Vwf*	*Klhl4*	*Hbegf*	*Egfl7*	*Gata6*	
Primitive Heart Tube	*Myh6*	*Ttn*	*Cnn1*	*Tnnt2*	*Myl4*	*Myh7*	*Myl7*		
Primitive Erythroid Lineage	*Hba-a1*	*HBB-y*	*Hba-x*	*Hbb-bh1*					
Definitive Erythroid Lineage	*Snca*	*Hbb-bs*	*Abcb4*	*Slc4a1*	*Kel*				

Table S1. Genes used for population identification

Table S2.

**Genotyping Primer**	**Sequence**	**Expected band sizes**
miR-302 forward	TTCACCCTCCGAGGACAGAA	735bp and 350bp wildtype and 260bp mutant
miR-302 wildtype reverse	CAGTTTAAGACACCCCCACTGA	
miR-302 mutant reverse	TAAGTGGCCACTGACACAGG	
miR-302 GFP forward	CAGGACCTACTTTCCCCAGAGCTG	274 bp wildtype and 547 bp GFP mutant
miR-302 GFP wildtype reverse	GAACCCACCCACAAGGCAACTAG	
miR-302 GFP mutant reverse	GAAGATGGTGCGCTCCTGGACGTAGC	
TD-tomato forward	CACTTGCTCTCCCAAAGTCG	550bp wildtype and 300bp mutant
TD-tomato wildtype reverse	TAGTCTAACTCGCGACACTG	
TD-tomato mutant reverse	GTTATGTAACGCGGAACTCC	
Cre 26	CCTGGAAAATGCTTCTGTCCG	300bp Gabra control and 400bp Cre
Cre 36	CAGGGTGTTATAAGCAATCCC	
Gabra 12	CAATGGTAGGCTCACTCTGGG AGATGATA	
Gabra 70	AACACACACTGGCAGGACTGGCTAGG	
Sox9 wildtype forward	GGGGCTTGTCTCCTTCAGAG	150bp wildtype and 200bp mutant
Sox9 flox forward	ACACAGCATAGGCTACCTG	
Sox9 reverse	TGGTAATGAGTCATACACAGTAC	
**Cloning Primer**	**Sequence**	
psicheck2_fwd	GGCTAGCATGCTGCAACTGCCAGCAGCATT CGAGCTGCAAACAAAGACTGAAAGCTAGTG	
psicheck2_rev	CTAGCTAGATCCGGTCCAGATCGATCTCAG AATTACTGCTCGTTCTTCAGCACGCGCTCCA CG	
UTR bs1 _fwd	GAGATCGATCTGGACCGGATCTAGCTAGAC ATGTAAAGGAAGGTAACGATTGCTGG	
UTR bs1 _rev	ATGCTGCTGGCAGTTGCAGCATGCTAGCCC GTCCTCCATGTTAACTCTGAAGGAGAC	
UTR bs1 _mut_fwd	GAGACTTGACGACTTTGTCTGAGC	
UTR bs1 _mut_rev	CAAAGTCGTCAAGTCTCTCTCCTGG	
UTR bs2 _fwd	GAGATCGATCTGGACCGGATCTAGCTAGCT ATCACCTGTACCTCCCTGAATACCAGCG	

UTR bs2 _rev	ATGCTGCTGGCAGTTGCAGCATGCTAGCCC ATAATTTAACAACAGATGACCATACCC	
UTR bs2 _mut_fwd	CTGTCTTGACGGTAGATTGTTGGC	
UTR bs2 _mut_rev	CAACAATCTACCGTCAAGACAGTAAA	
UTR bs3 _fwd	GAGATCGATCTGGACCGGATCTAGCTAGGA TAGCCCCTTGGCTGCTCTCCTGCAGAG	
UTR bs3 _rev	ATGCTGCTGGCAGTTGCAGCATGCTAGCCC GAAAGATGCCATATTATAAGAACGC	
UTR bs3 _mut_fwd	CTTTTTTTTTTTGACGACAGCAGTC	
UTR bs3 _mut_rev	CTGCTGTCCTGAAAAAAAAAAAGAAAGAAAG	

Table S2. PCR primers and expected band sizes

Supplementary References:

75. B. Pijuan-Sala et al., A single-cell molecular map of mouse gastrulation and early organogenesis. Nature 566, 490–495 (2019).
76. C. Trapnell et al., The dynamics and regulators of cell fate decisions are revealed by pseudotemporal ordering of single cells. Nat. Biotechnol. 32, 381 (2014).
77. X. Qiu et al., Single-cell mRNA quantification and differential analysis with Census. Nat. Methods 14, 309–315 (2017).
78. X. Qiu et al., Reverse graph embedding resolves complex single-cell developmental trajectories (2017). https://www. biorxiv. org/content/10.1101/110668v1. Accessed January 17th 2020.

78. X. Qiu et al., Reverse graph embedding resolves complex single-cell developmental trajectories (2017). https://www. biorxiv. org/content/10.1101/110668v1. Accessed January 17th 2020.

79. B. P. Lewis, C. B. Burge, D. P. Bartel, Conserved seed pairing, often flanked by adenosines, indicates that thousands of human genes are microRNA targets. Cell 120, 15–20 (2005).
80. Z. E. Stine et al., Oligodendroglial and pan-neural crest expression of Cre recombinase directed by Sox10 enhancer. Genesis 47, 765–770 (2009).
81. H. Luche, O. Weber, T. N. Rao, C. Blum, H. J. Fehling, Faithful activation of an extra-bright red fluorescent protein in “knock-in” Cre-reporter mice ideally suited for lineage tracing studies. Eur. J. Immunol. 37, 43–53 (2007).
82. Y. Xia et al., T5 exonuclease-dependent assembly offers a low-cost method for efficient cloning and site-directed mutagenesis. Nucleic Acids Res. 47, e15 (2019).
83. A. Repele, Robust normalization of luciferase reporter data. Methods Protoc. 2, 62 (2019).
